# Comparative effectiveness of antimicrobial implant surface coatings in preventing orthopaedic implant-associated infections: a network meta-analysis

**DOI:** 10.1007/s00402-026-06225-3

**Published:** 2026-02-10

**Authors:** Randa Elsheikh, Abdelrahman Makram, László Toth, Michael Hirschmann, Matthew Adam

**Affiliations:** 1 University Department of Orthopedic Surgery and Traumatology, Kantonsspital Baselland, CH-4101 Bruderholz, Switzerland., Basel, Switzerland; 2 Deanery of Biomedical Sciences at Edinburgh Medical School, University of Edinburgh, Edinburgh, United Kingdom., University of Edinburgh, Edinburgh, UK; 3 School of Public Health, Imperial College London, London, UK; 4 Biomechanics and Implant Design, Department of Biomedical Engineering, University of Basel, Basel, Switzerland; 5 Department of Clinical Research, Research Group Michael T. Hirschmann, Regenerative Medicine & Biomechanics, University of Basel, Basel, Switzerland; 6 Clinical Infection Research Group, NHS Lothian, Edinburgh, UK; Deanery of Clinical Sciences, University of Edinburgh, Edinburgh, UK

**Keywords:** Implant-associated infection, Prosthetic joint infection, Fracture-related infection, Antimicrobial implant coating, Local antibiotic carrier, DAC-hydrogel, Gentamicin, Silver, Iodine

## Abstract

**Purpose:**

Implant-associated infections (IAIs) remain a major challenge in orthopaedic surgery, causing substantial morbidity, mortality, and healthcare costs. Antimicrobial implant coatings have emerged as a promising preventive strategy, but their comparative clinical benefit remains unclear. This study aimed to evaluate the effectiveness of antimicrobial coatings in preventing IAIs and to compare their clinical performance to uncoated implants.

**Methods:**

A systematic review and network meta-analysis was conducted in accordance with PRISMA guidelines. Medline, Embase, Scopus, and Web of Science were systematically searched for comparative studies evaluating antimicrobial implant coatings for the prevention of orthopaedic IAIs. The primary outcome was the incidence of postoperative IAIs, while secondary outcomes included complications, site-specific infections, causative organisms, use of antibiotic prophylaxis, operative time, time to infection, and implant survival. Random-effects network meta-analysis, subgroup analyses, and assessment of publication bias were performed to synthesize and compare treatment effects across coating types.

**Results:**

Twenty-six studies encompassing 3,592 patients were included, of whom 1,576 received coated and 2,016 uncoated implants. Coating technologies included Defensive Antibacterial Coating (DAC) hydrogel, gentamicin, iodine, silver, antibiotic-loaded calcium sulfate, and gold-silver-palladium alloy. Overall, infection rates were lower in coated implants (26.9% vs. 73.1%). Network meta-analysis demonstrated that DAC-hydrogel (OR = 0.10, 95% CI: 0.03–0.28, *p* < 0.001), gentamicin (OR = 0.27, 95% CI: 0.09–0.80, *p* = 0.018), iodine (OR = 0.34, 95% CI: 0.12–0.95, *p* = 0.039), and silver (OR = 0.67, 95% CI: 0.48–0.95, *p* = 0.026) significantly reduced infection risk. Coated implants were also associated with fewer postoperative complications (OR = 0.28, 95% CI: 0.09–0.85, *p* = 0.025), delayed infection onset (IRR = 0.24, 95% CI: 0.06–0.95, *p* = 0.042), and no increase in operative time.

**Conclusion:**

There is enough statistical evidence to suggest that antimicrobial implant coatings may reduce implant-associated infections and postoperative complications without increasing operative time. High-dose local antibiotic carriers, such as DAC-hydrogel and gentamicin, are associated with the largest reductions in infection risk, supporting their potential protective role in high-risk procedures.

**Supplementary Information:**

The online version contains supplementary material available at 10.1007/s00402-026-06225-3.

## Introduction

Implant-associated infections (IAIs) are one of the most serious complications in orthopaedic surgery. Despite adherence to aseptic surgical protocols and the routine use of systemic antibiotic prophylaxis, it is estimated that prosthetic joint infections (PJIs) affect up to 15% and 36% of patients undergoing primary and revision arthroplasty and tumor endoprostheses, respectively [1–3]. Similarly, fracture-related infections (FRIs) are reported in up to 2% of all closed fractures and 30% of complex open tibia fracture fixation [4]. The economic burden of PJIs alone has been estimated to amount to an average of €30,000 per case [5], with an annual cost of approximately €350 million [6] and $566 million [7] in Europe and the United States, respectively, and a projected cost of $1.85 billion by 2030 [8].

A key challenge in preventing IAIs lies in the pathogenesis of the infection itself. The sterile implant surface is rapidly conditioned by host proteins, which, despite facilitating tissue integration, present ligands recognized by bacterial adhesins [9]. Although early bacterial attachment is reversible, it typically progresses to biofilm formation within hours [10, 11]. Subsequent secretion of an extracellular polymeric substance stabilizes the biofilm [9] and confers substantial protection against host immune mechanisms and systemic antibiotics [12]. As the biofilm matures and local nutrients decline, microbial cells begin to gradually disperse, enabling colonization of adjacent tissues and propagation of infection [9].

To minimize the risk of IAIs, a combination of systemic and local prophylactic measures is employed. Systemic antibiotic prophylaxis, typically administered within one hour of surgical incision, aims to reduce the microbial load in the bloodstream and surrounding tissues at the time of implantation. While effective in reducing early postoperative infections, their subtherapeutic concentrations at the implant interface may not be enough to prevent bacterial surface adherence, especially due to the poor surgical site penetration in the ischemic post-operative tissue [1]. Similarly, to intercept the bacterial race for the surface [11] and prevent biofilm formation, the use of locally delivered antibiotics was introduced in the late 1970 s [13]. This paved the road for the use of antibiotic-impregnated polymethylmethacrylate (PMMA) cement in total joint arthroplasty [12, 14]. Nonetheless, the effect of most standard local antibiotics in soft tissues lasts only up to 72 h, after which the implant becomes vulnerable to bacterial colonization [15, 16]. The application of antibiotics on cement has also been shown to promote cement degradation, and once antibiotics have been released, the PMMA becomes an inert porous material, constituting a potential niche for bacterial colonization and infection [17].

A critical limitation of all these strategies is that none directly modify the implant surface, which is the primary site of bacterial colonization and biofilm formation. Consequently, antimicrobial implant coatings have been developed as a potential method of prevention and treatment of IAIs [18]. These technologies operate through different mechanisms. Passive surface modifications (PSMs) alter the physicochemical properties of the implant to reduce bacterial adhesion without releasing bactericidal agents. Active surface modifications (ASM), such as silver and iodine, integrate pharmacologically active antibacterial compounds within the implant surface, enabling direct antimicrobial activity. Peri-operative local antibiotic carriers (LACs) deliver high local concentrations of pre-loaded antibiotics in the peri-implant tissues or enhance the activity of systemic antibiotics [18]. Examples of LACs include Defensive Antibacterial Coating (DAC) hydrogel and antibiotic-loaded calcium sulfate (ALCS), which, unlike traditional calcium sulfate beads, is preloaded with antibiotics for controlled release and can be molded for direct application to the implant surface.

Despite their theoretical benefits, the clinical effectiveness of antimicrobial surface coatings remains incompletely defined. Importantly, previous studies have failed to assess how different coating materials compare to each other [1], where only a few head-to-head trials have been performed, with available data being dispersed across small or single-center studies. As such, there is weak evidence to guide implant selection, particularly when balancing infection prevention with biocompatibility and cost.

This study aimed to evaluate the effectiveness of antimicrobial implant coatings in preventing orthopaedic IAIs and to compare the relative performance of different coating technologies, as well as uncoated implants, in terms of postoperative infection rates.

It was hypothesized that antimicrobial implant coatings would significantly reduce the risk of IAIs compared with uncoated implants.

## Methods

### Search strategy

This study was conducted following the Preferred Reporting Items for Systematic Reviews and Meta-Analysis (PRISMA) guidelines [19]. The protocol was prospectively registered on PROSPERO under the registration number CRD42024608784.

An electronic search was undertaken in Medline, Embase, Scopus, and Web of Science from their inception to November 2024 using a combination of string terms related to antimicrobial agents (e.g., silver, iodine, antibiotics), orthopaedic implants (e.g., nail, plate, prosthesis), and implant-associated infections (e.g., periprosthetic joint infection, fracture-related infection). The full search strategy is outlined in Supplementary Table 1. A manual search was performed in April and repeated in October 2025.

### Eligibility criteria and study selection

Studies were included if they assessed the use of antimicrobial surface implant coatings in preventing orthopaedic IAIs, if comparative, and if performed on humans. There were no restrictions on the type of orthopaedic procedure, publication date, or follow-up period. Craniofacial implants (including dental implants), case reports, case series, review articles, letters to the editor, opinion articles, in vitro studies, in vivo animal studies, studies evaluating antibiotic-impregnated PMMA, and non-English studies were excluded.

Title and abstract screening and full-text screening were performed independently by two reviewers. Any discrepancy during the screening process was resolved by consensus. Weighted Kappa () scores were used to assess agreement between reviewers at each phase of the screening process. A priori agreement was categorized as slight (0.01 to 0.20), moderate (0.41 to 0.60), substantial (0.61 to 0.80), and high (0.81 to 0.99) [20].

### Data extraction

Data extraction was conducted by one reviewer and verified by a second using Covidence (Veritas Health Innovation, Melbourne, Australia). A standardized, a priori-developed extraction form captured study characteristics, population details, intervention and comparator specifics, and outcomes. In case of missing or unclear data, study authors were contacted for clarification.

### Outcome measures

The primary outcome was the incidence of postoperative IAIs. Secondary outcomes included complications requiring surgery, operation time, time to infection, and infection stratified by anatomical site, device type, organism type, resistance pattern, and use of antibiotic prophylaxis.

### Quality assessment

The methodological quality and risk of bias of the included studies were independently assessed by two reviewers. For randomized controlled studies (RCTs), the Revised Cochrane risk-of-bias tool (RoB 2) was applied, whereas for observational studies, the Risk of Bias in Non-randomized Studies – of Intervention (ROBINS-I) tool was used [21, 22]. The RoB 2 tool consists of five domains: bias arising from the randomization process, deviation from intended interventions, missing outcome data, measurement of outcomes, and selection of the reported result. Each domain is judged as having a low risk of bias, some concerns, or a high risk of bias, with an overall risk of bias determined by combining the scores from the five domains. In the ROBINS-I tool, seven domains are assessed: bias due to confounding, participant selection, intervention classification, deviation from intended interventions, missing data, outcome measurement, and selective reporting. Each domain is scored as low, moderate, serious, or critical risk of bias, with an overall bias determined by the combination of all the scores assigned to each domain.

### Statistical analysis

Several frequentist network meta-analyses (NMAs) were performed using the *netmeta* package (version 3.1-1.1; based on *meta* version 8.0–2)^23^ within R software (version 4.4.3; R Foundation for Statistical Computing; Vienna, Austria) [23]. Effect estimates were expressed as odds ratios (ORs) for binary outcomes, mean differences (MDs) for continuous outcomes, and incidence rate ratios (IRRs) for time-to-event outcomes, each with corresponding 95% confidence intervals (CIs). All analyses used random-effects models, with non-coated implants serving as the common reference comparator.

Relative treatment rankings were derived using P-scores (0–1 scale, higher indicating better performance) [24, 25]. League tables and network plots illustrated pairwise comparisons and overall network structure. Consistency between direct and indirect evidence was evaluated using the *netsplit* approach. Funnel plots and Egger’s test were used to evaluate small-study effect when ≥ 10 studies were available [26, 27].

Subgroup NMAs were performed for the postoperative infection based on anatomical site, causative organism, implant type, antibiotic resistance, and use of systemic antibiotic prophylaxis.

Results were considered statistically significant if the two-sided p-value was less than 0.05 and the 95% CI excluded the null value.

## Results

### Search results

**Fig. 1:**
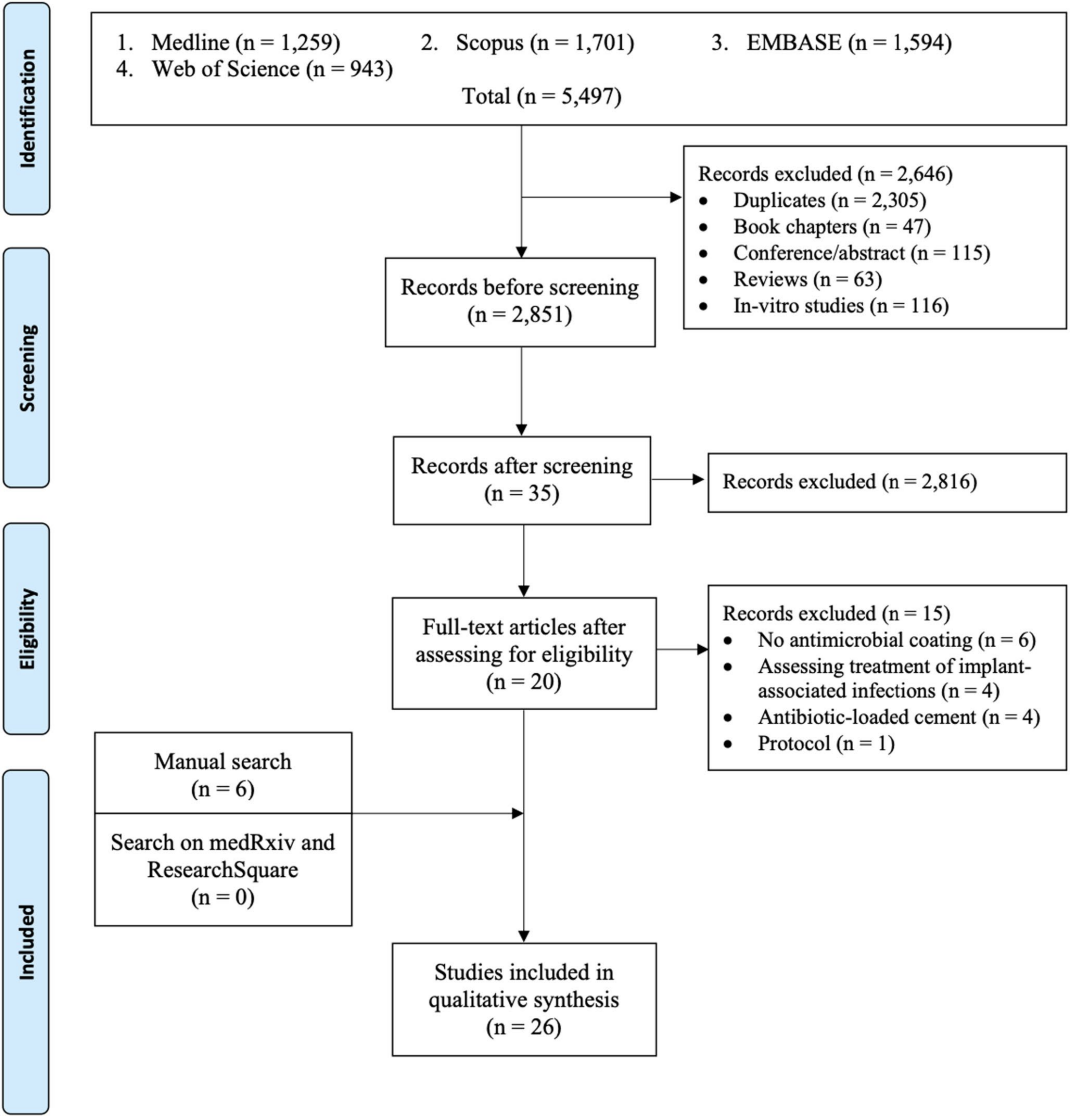
PRISMA flow diagram showing the study selection process

A total of 2,851 studies were identified during the initial literature search. Following title, abstract, and full-text screening, 20 studies met the eligibility criteria. Six additional studies were identified through cross-referencing, resulting in 26 included studies (Fig. 1). The agreement was moderate for title and abstract screening (=0.60) and high for full-text screening (=0.89, 95% CI: 0.74–1.00). Of the included studies, three were RCTs, and 23 were observational.

### Baseline characteristics of the included studies

3,592 patients were included in the final analysis, of whom 1,576 (43.87%) received antimicrobial-coated implants and 2,016 (56.1%) uncoated implants. The most commonly used coating technologies were silver, DAC-hydrogel, ALCS, and iodine used in 648 (41.1%), 521 (33%), 111 (7%), and 104 (6.6%) of the cases, respectively.

Nineteen studies (2,258 patients) reported sex distribution: 1,141 (50.5%) males, and 1,117 (49.4%) females (Table 1). The mean age was slightly higher in the coated group (60.6 ± 22.0 years) compared with the uncoated group (55.4 ± 33.2 years), whereas BMI was comparable between groups (26.3 ± 5.9 vs. 25.7 ± 6.2 kg/m^2^). The mean follow-up was 28.6 ± 34.4 months in the coated group versus 36.6 ± 38.9 months in the uncoated group.

**Table 1:** Baseline characteristics of the included studies

Author, Year	Country	Study Design	Evidence Level	Implant Type	Coating Technology	Sample Size *n* (%)			Sex *n* (%)		Age (mean±SD)	Follow-up (mean±SD; mths)
						Total	Coated	Uncoated	Male	Female		
Hardes et al., 2010[28]	Germany	Prospective cohort	II	Tumour endoprosthesis (MUTARS^®^, Implantcast, Germany)	Silver	125	51 (40.8%)	74 (59.2%)	NR	NR	NR	19 ± 15
Wafa et al., 2015 [28]	UK	Retrospective case-control	III	Agluna, silver-enhanced (Accentus Medical Ltd, UK) custom-made endoprosthesis (Stanmore Implants Worldwide, UK)	Silver	170	85 (50%)	85 (50%)	106 (62.3%)	64 (37.6%)	42.2 ± 53.83	NR
Donati et al., 2016 [29]	Italy	Retrospective case-control	III	Tumour endoprosthesis (MUTARS^®^, Implantcast, Germany)	Silver	68	38 (55.8%)	30 (44.1%)	31 (45.6%)	37 (54.4%)	61.61 ± 40.46	46.5 ± 24
Piccioli et al., 2016 [30]	Italy	Retrospective case-control	III	Tumour endoprosthesis (MUTARS^®^, Implantcast, Germany)	Silver	30	17 (56.6%)	13 (43.4%)	14 (46.6%)	16 (53.3%)	56.20 ± 48.89	38.8 ± 21.5
Romano et al., 2016 [31]	Italy	Randomized controlled trial	II	Total joint prosthesis	DAC-hydrogel	373	189 (50.7%)	184 (49.3%)	155 (41.5%)	118 (31.6%)	70.01 ± 11.68	14.5 ± 5.5
Shirai et al., 2016 [32]	Japan	Retrospective case-control	III	Osteosynthesis devices and joint prosthesis	Iodine	100	38 (38%)	62 (62.0%)	39 (39%)	23 (23%)	31.9 ± 2.3	32.1 ± 3
Hardes et al., 2017 [33]	Germany	Prospective cohort	II	Tumour endoprosthesis (MUTARS^®^, Implantcast, Germany)	Silver	98	56 (57.1%)	42 (42.8%)	NR	NR	NR	57.3 ± 60.3
Malizos et al., 2017 [34]	Greece	Randomized controlled trial	II	Osteosynthesis devices (Stryker Inc., USA; Smith-Nephew, UK; DePuy-Synthes, USA)	DAC-hydrogel	253	126 (49.8%)	127 (50.2%)	110 (43.5%)	143 (56.5%)	60.54 ± 19.53	18.1 ± 4.5
Zajonz et al., 2017 [35]	Germany	Retrospective cohort	III	Tumour endoprostheses (MUTARS^®^, Implantcast, Germany; MML München-Lübeck^™^, AQ Implants, Germany)	Silver	34	20 (58.8%)	14 (41.17%)	15 (44.1%)	19 (55.8%)	66.02 ± 35.04	104 ± 64
Medellin et al., 2019 [36]	United Kingdom	Retrospective cohort	III	Tumour endoprosthesis (Stanmore Implants Worldwide, UK)	Silver	81	23 (28.4%)	58 (71.6%)	43 (53.1%)	38 (46.9%)	43 ± 55.85	123.6 ± 90.6
Miwa et al., 2019 [37]	Japan	Retrospective case-control	III	Osteosynthesis devices	Iodine	302	66 (21.8%)	236 (78.1%)	173 (57.3%)	129 (42.7%)	43.1 ± 65.55	NR
Parry et al., 2019 [38]	United Kingdom	Retrospective case-control	III	Agluna, silver-enhanced (Accentus Medical Ltd, UK) custom-made endoprosthesis (Stanmore Implants Worldwide, UK)	Silver	394	89 (22.6%)	305 (77.4%)	NR	NR	NR	54.9 ± 29.8
Pinto et al., 2019 [39]	India	Retrospective case-control	III	Intramedullary nail (Matrix Meditec Pvt, India)	Gentamicin	28	14 (50%)	14 (50%)	NR	NR	NR	NR
Sambri et al., 2019 [40]	Italy	Retrospective case-control	III	Tumor endoprosthesis	Silver	55	20 (36.3%)	35 (63.6%)	27 (49.1%)	28 (50.9%)	35.77 ± 39.52	NR
Streitbuerger et al., 2019 [41]	Germany	Prospective cohort	II	Tumour endoprosthesis (MUTARS^®^, Implantcast, Germany)	Silver	99	64 (64.6%)	35 (35.3%)	NR	NR	39.62 ± 52.40	61.4 ± 46.7
Zagra et al., 2019 [42]	Italy	Retrospective case-control	III	Total joint prosthesis	DAC-hydrogel	54	27 (50%)	27 (50%)	NR	NR	NR	32.4 ± 4.2
De Meo et al., 2020 [43]	Italy	Retrospective case-control	III	Osteosynthesis devices and joint prosthesis	DAC-hydrogel	34	17 (50%)	17 (50%)	13 (38.2%)	21 (61.7%)	75.40 ± 10.59	23.3 ± 18.9
Sambri et al., 2020 [44]	Italy	Retrospective case-control	III	Tumour endoprosthesis (Megasystem C^®^, Waldemar Link, Germany)	Silver	68	29 (42.6%)	39 (57.3%)	37 (54.4%)	31 (45.6%)	29.41 ± 46.22	56.4 ± 44.1
Greco et al., 2021 [45]	Italy	Retrospective case-control	III	Intramedullary nail (ETN PROtect and ETN—Expert Tibial Nail, DePuy-Synthes, USA)	Gentamicin	46	23 (50%)	23 (50%)	37 (80.4%)	9 (19.5%)	43.45 ± 18.36	NR
Zoccali et al., 2021 [46]	Italy	Retrospective case-control	III	Tumour endoprostheses (GMRS^™^, Global Modular Replacement System, Stryker, USA; MUTARS^®^, Implantcast, Germany; custom-made prostheses, Adler Ortho S.P.A. and MT-Ortho S.R.L., Italy)	DAC-hydrogel	86	43 (50%)	43 (50%)	44 (51.1%)	38 (44.2%)	46.50 ± 20.42	24.2 ± 11.5
Pala et al., 2022 [47]	Italy	Retrospective cohort	III	Tumour endoprosthesis (MUTARS^®^, Implantcast, Germany)	Silver	187	118 (63.1%)	69 (36.9%)	100 (53.5%)	87 (46.5%)	53 ± 63.50	40.8 ± 47.8
Sacchetti et al., 2022 [48]	Italy	Retrospective case-control	III	Tumour endoprosthesis (Megasystem C^®^, Waldemar Link, Germany)	Silver	142	38 (26.7%)	104 (73.23%)	54 (38.0%)	88 (62%)	61 ± 21.9	NR
McPherson et al., 2024 [49]	US	Retrospective case-control	III	Total joint prosthesis (Zimmer-Biomet, USA)	ALCS	205	111 (54.1%)	104 (50.7%)	102 (49.7%)	113 (55.1%)	66.1 ± 50.77	56.6 ± 18
Ding et al., 2025 [50]	Italy	Retrospective cohort	III	Total joint prosthesis	DAC-hydrogel	238	119 (50%)	119 (50%)	115 (48.3%)	119 (50%)	68.30 ± 11.50	44.4 ± 23.2
Rai et al., 2025 [51]	India	Randomized controlled trial	II	Intramedullary nail (Matrix Meditec Pvt, India)	Gentamicin	124	62 (50%)	62 (50%)	81 (65.3%)	53 (42.7%)	37 ± 3.42	NR
Wiechert et al., 2025 [52]	Germany	Retrospective cohort	III	Intramedullary nail (Bactiguard, Zimmer-Biomet, USA)	GSP alloy	188	93 (49.4%)	95 (50.5%)	72 (38.3%)	116 (61.7%)	83.66 ± 13.77	NR

NR: Not reported; DAC: Defensive antimicrobial coating; ALCS: Antibiotic-loaded calcium sulfate; GSP: Gold, silver, palladium

Regarding implant type, 14 studies assessed antimicrobial coatings in tumor endoprostheses [28–30, 33, 35, 36, 38, 40, 41, 44, 46–48, 53], seven in osteosynthesis devices [32, 34, 37, 39, 45, 51, 52], and five in primary and revision joint arthroplasty [31, 42, 43, 49]. Across all studies, 291 (8.1%) patients experienced a previous IAI, and 553 (15.4%) underwent a prior revision surgery, of which one-half were single-stage and the other half two-stage.

### Risk of bias and quality assessment

**Fig. 2:**
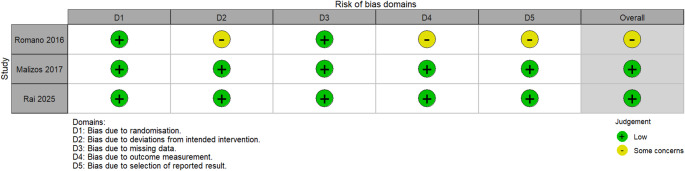
Overall risk of bias in the included interventional studies assessed using the Cochrane RoB2 tool. Green represents a low risk of bias and yellow represents some concerns

**Fig. 3:**
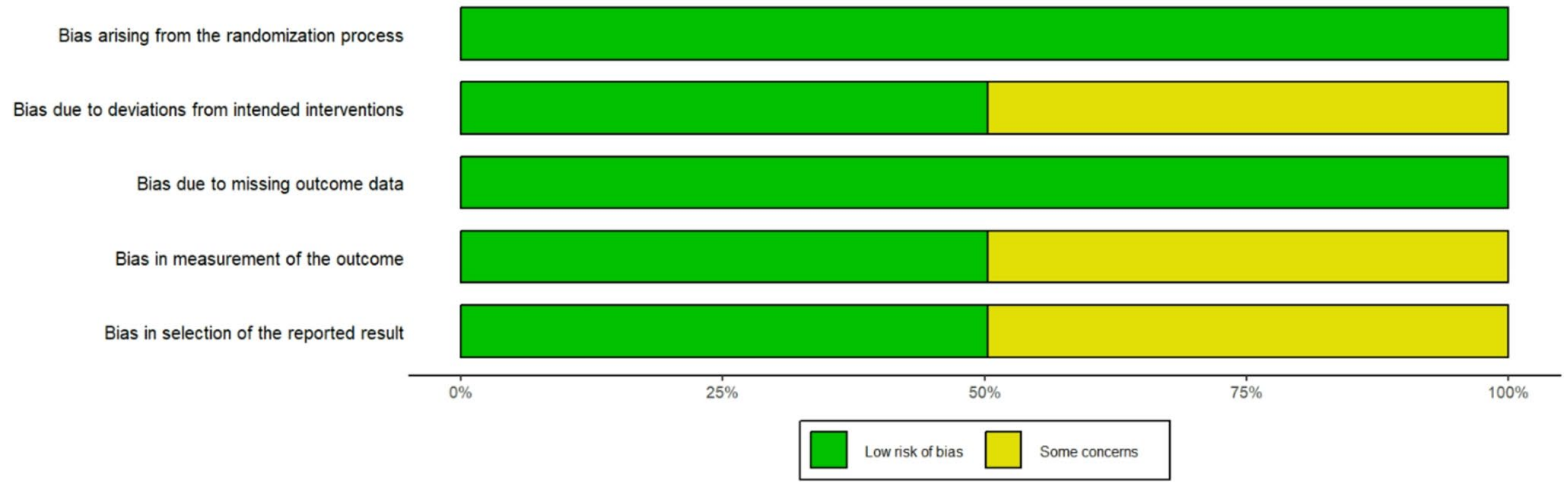
Domain-specific risk of bias of the included interventional studies assessed using the Cochrane RoB2 tool. Green represents a low risk of bias and yellow represents some concerns

Among randomized trials, most domains were rated at low risk of bias, though one trial [31] had some concerns regarding deviations from intended interventions, missing outcome data, and selective reporting (Figs. 2 and 3).

**Fig. 4:**
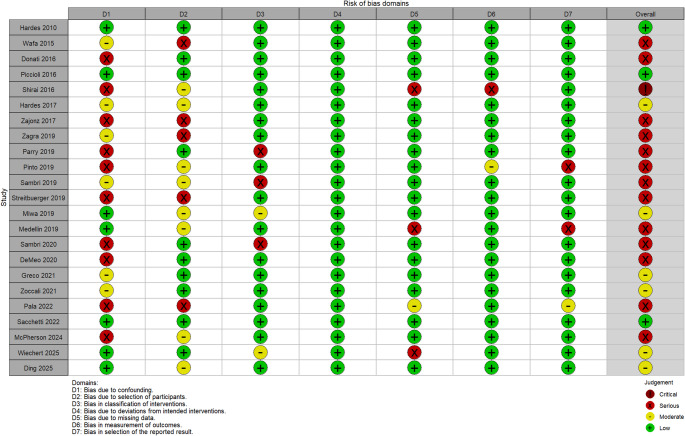
Overall risk of bias in the included non-interventional studies assessed using the Cochrane ROBINS-I tool. Green represents a low risk of bias, yellow represents moderate risk, light red represents serious risk, and dark red represents critical risk

**Fig. 5:**
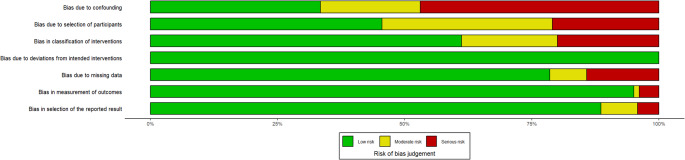
Domain-specific risk of bias of the included non-interventional studies assessed using the Cochrane ROBINS-I tool. Green represents a low risk of bias, yellow represents moderate risk, light red represents serious risk, and dark red represents critical risk

Non-randomized studies frequently had moderate to serious risk of bias, mainly due to confounding, patient selection, intervention classification, missing data, and selective outcome reporting. Several studies lacked clarity on confounder adjustment, participant selection, and timing of intervention (Figs. 4 and 5).

Overall, only five studies were considered high quality and at low risk of bias [30, 34, 48, 50, 51, 53]. Despite these limitations, all studies were retained to preserve the comprehensiveness of the evidence base.

### Study and intervention characteristics

#### Definition of implant-associated infection

Definitions of IAI varied greatly across the included studies. Seven studies [33, 38, 40, 41, 44, 48] applied the Musculoskeletal Infection Society (MSIS) definition [54], four [35, 42, 46, 49] adopted the International Consensus Meeting (ICM) Criteria [55], and five [28, 34, 37, 45, 53] followed the Centers for Disease Control and Prevention (CDC) definitions, distinguishing superficial, deep incisional, and organ/space infections [56]. One study [52] applied the Metsemakers criteria [57] for FRIs, and another [43] used the European Bone and Joint Infection Society (EBJIS) definition [58]. The remaining studies [29–31, 39, 47, 51] used study-specific combinations of clinical, laboratory, and microbiological parameters, whereas two studies [32, 36] failed to report their diagnostic criteria.

#### Coating technology

Thirteen studies [28–30, 33, 35, 36, 38, 40, 41, 44, 47, 48, 53] assessed the use of silver-based coatings, either in the ionically active [28, 38] or elementary form [33, 41, 53]. DAC-hydrogel was used in six studies [31, 34, 42, 43, 46, 50], and was prepared following the manufacturer’s recommendations [59]. Gentamicin-coated poly-D, L-lactide (PDDLA), which deploys gradual release of antibiotics followed by PDDLA hydrolytic degradation, was used in three studies [39, 45, 51]. Iodine-based coatings were used in two studies [32, 37], with only one study reporting the used preparation [32]. Other coating materials included gold, silver, palladium (GSP) noble metal alloy (Bactiguard^®^)^42^ and ALCS [49].

Antibiotic selection for DAC-hydrogel and ALCS was tailored to the clinical setting and local susceptibility patterns.

### Outcomes

#### Implant-associated infections

A total of 346 IAIs were reported across the included studies, with a higher incidence in uncoated implants (73.1%) than in coated ones (26.9%) (Supplementary Table 2). Overall, early infections (< 3 months) predominated, particularly in uncoated implants (84.2% vs. 10.5%), whereas late infections were slightly higher in the coated group (57.1% vs. 42.8%).

**Fig. 6:**
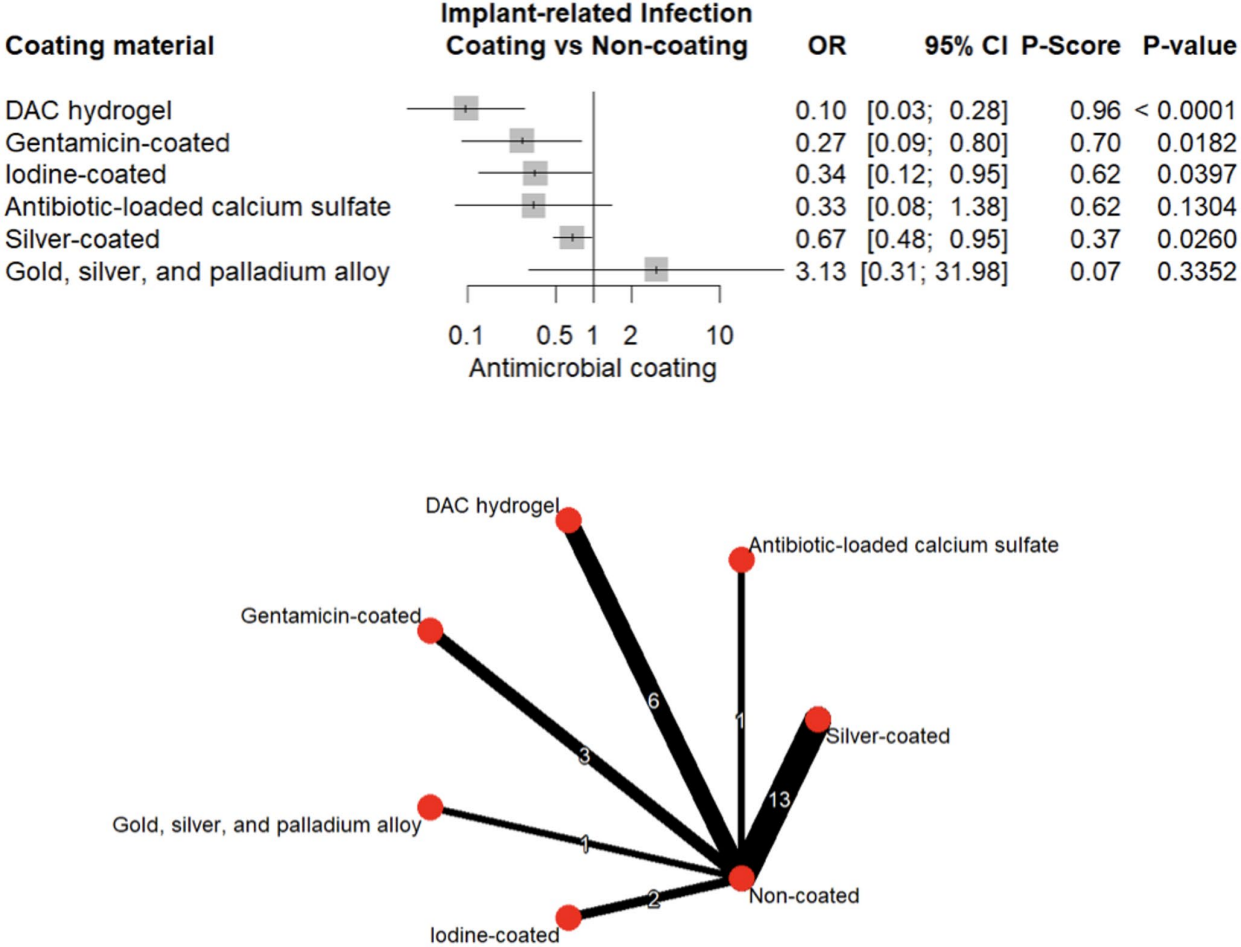
Forest plot and network graph illustrating the OR and 95% CIs for implant-associated infections, with ranked P-scores

**Fig. 7:**
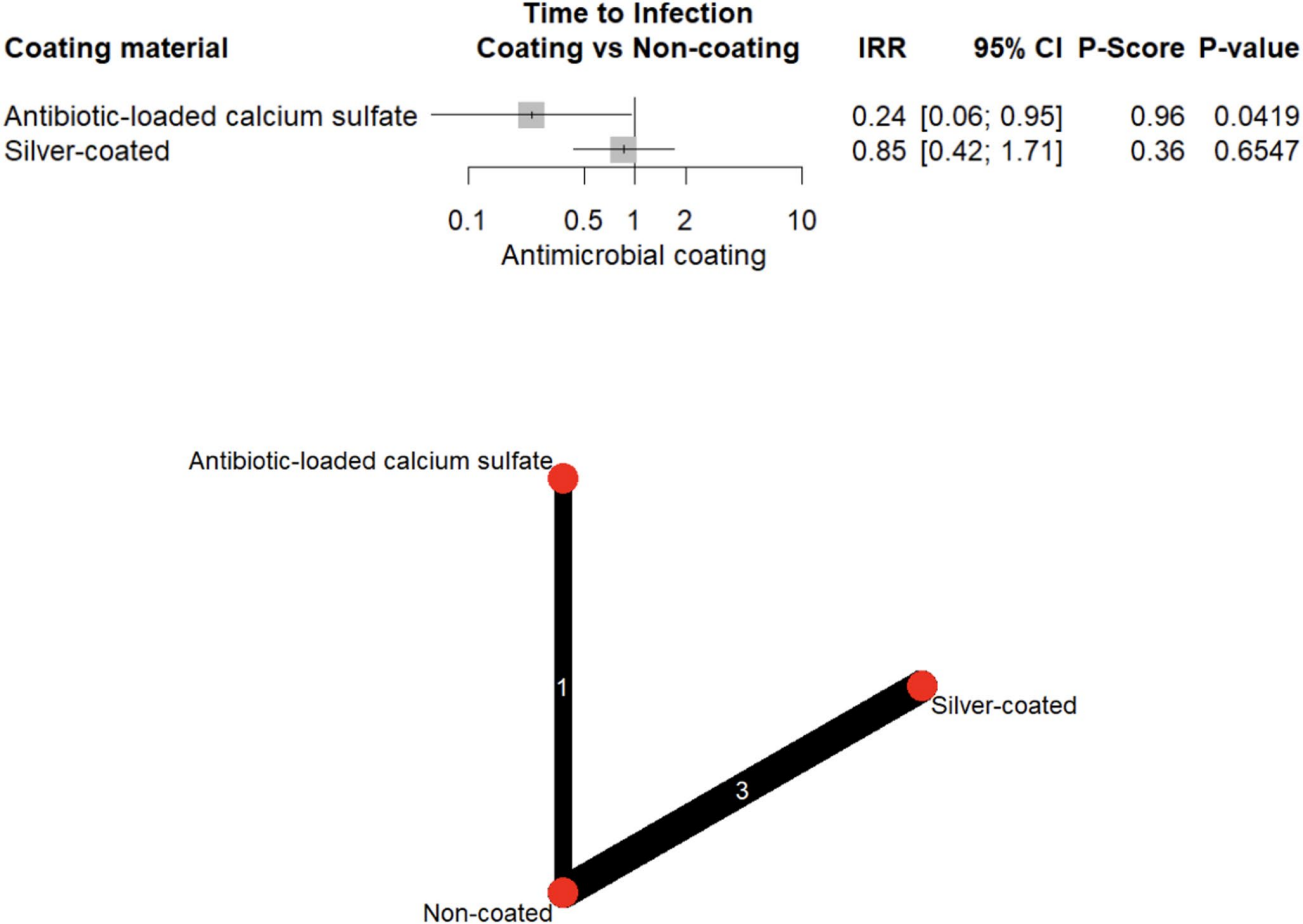
Forest plot and network graph illustrating the IRR and 95% CIs for time to infection, with ranked P-scores

Pooled analysis demonstrated significantly lower odds of infection with several antimicrobial coatings, including DAC-hydrogel (OR = 0.10, 95% CI: 0.03–0.28, *p* < 0.001), gentamicin (OR = 0.27, 95% CI: 0.09–0.80, *p* = 0.018), iodine (OR = 0.34, 95% CI: 0.12–0.95, *p* = 0.039), and silver (OR = 0.67, 95% CI: 0.48–0.95, *p* = 0.026). In contrast, ALCS and GSP alloy did not exhibit a significant protective effect (*p* > 0.05) (Fig. 6), although ALCS was associated with a significant delay in infection onset (IRR = 0.24, 95% CI: 0.06–0.95, *p* = 0.042) (Fig. 7). Evidence of publication bias was detected (Egger’s test, *p* = 0.014; Supplementary Fig. 1).

When ranking coating technologies by estimated effectiveness, DAC-hydrogel exhibited the highest protective effect (P-score = 0.96), followed by gentamicin (0.70). Iodine and ALCS demonstrated moderate effectiveness (P-score = 0.62 each), whereas data for silver (0.37) and GSP alloy (0.07) suggested lower clinical impact (Fig. 6). These rankings reflect relative performance across analyzed studies and should not be interpreted as absolute hierarchies.

Among infection cases, Gram-positive organisms were the most isolated pathogens, particularly in uncoated implants (20.2% vs. 9.5%). No significant differences were found in pathogen-specific infection rates (*p* > 0.05). However, DAC-hydrogel (OR = 0.08, 95% CI: 0.02–0.36, *p* < 0.001), gentamicin (OR = 0.27, 95% CI: 0.09–0.79, *p* = 0.016), and iodine (OR = 0.35, 95% CI: 0.13–0.93, *p* = 0.035) were associated with significantly lower rates of infections of unknown origin (Supplementary Fig. 2). None of the coatings, however, conferred additional protection against resistant organisms.

#### Surgical site

A total of 2,132 procedures were analyzed, 91.8% involving the lower limb and 8.2% the upper limb. The femur was the most frequently treated site (44.3%), followed by the tibia and hemipelvis. In the upper limb, procedures were primarily performed in the humerus (85.8%), with a few involving the clavicle, forearm, and hand.

**Fig. 8:**
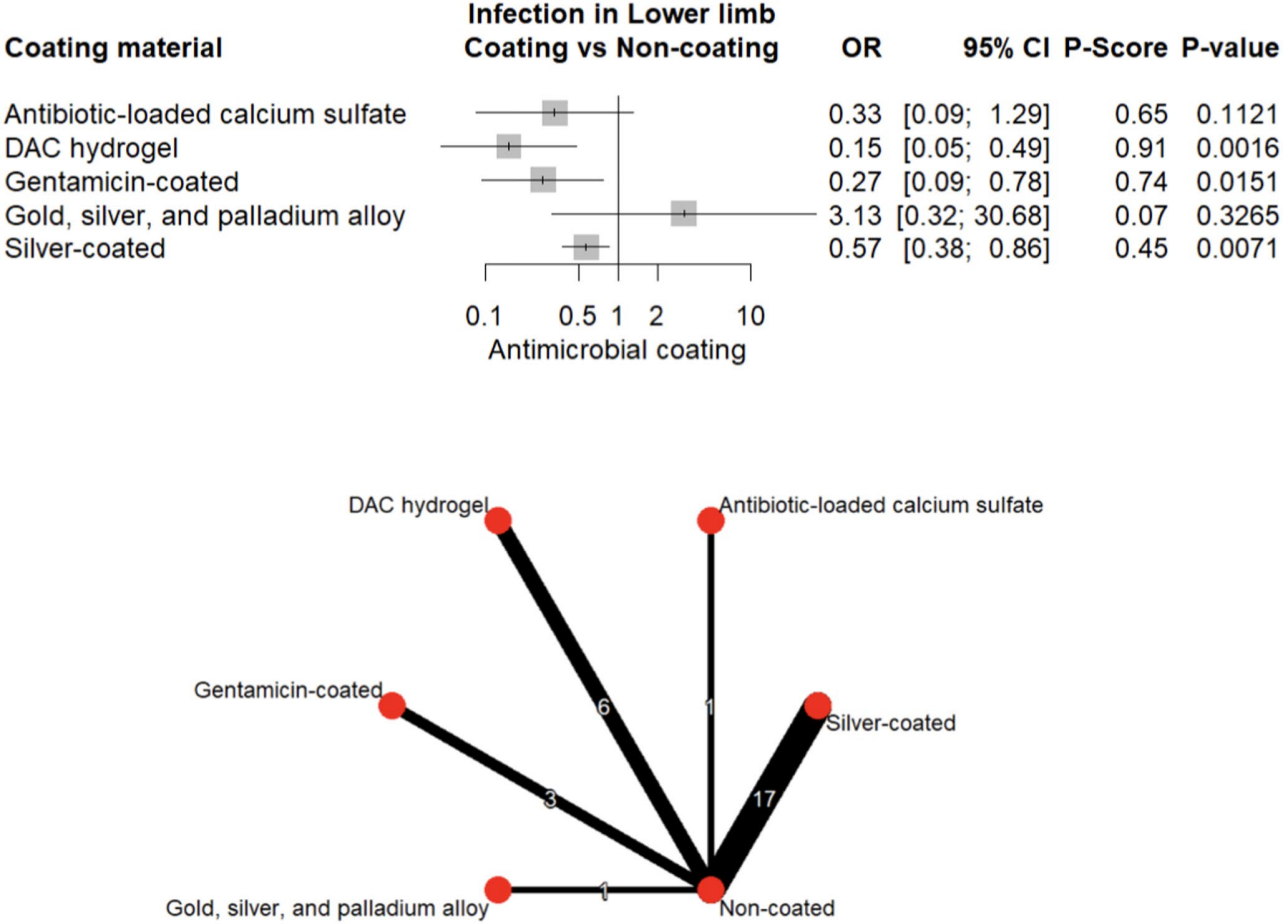
Forest plot and network graph illustrating the OR and 95% CIs for implant-associated infections occurring in the lower-limb, with ranked P-scores

Antimicrobial coatings showed no significant effect in upper limb reconstructions (*p* > 0.05). In contrast, significant reduction in infection rates was observed in the lower limb, with DAC-hydrogel, gentamicin, and silver achieving 85% (95% CI: 51–95%, *p* = 0.001), 73% (95% CI: 22–91%, *p* = 0.015), and 43% (95% CI: 14–62%, *p* = 0.007) infection reduction, respectively (Fig. 8). Site-specific analysis revealed that DAC-hydrogel was particularly effective in femoral reconstructions (OR = 0.08, 95% CI: 0.01–0.67, *p* = 0.020), while gentamicin (OR = 0.27, 95% CI: 0.09–0.84, *p* = 0.023) and silver (OR = 0.49, 95% CI: 0.24–1.00, *p* = 0.049) showed additional benefit in the tibia.

#### Prosthesis type

Coating materials were used in 45.3% of the 2,551 prostheses and 49.8% of the 639 osteosynthesis devices assessed in the study.

**Fig. 9:**
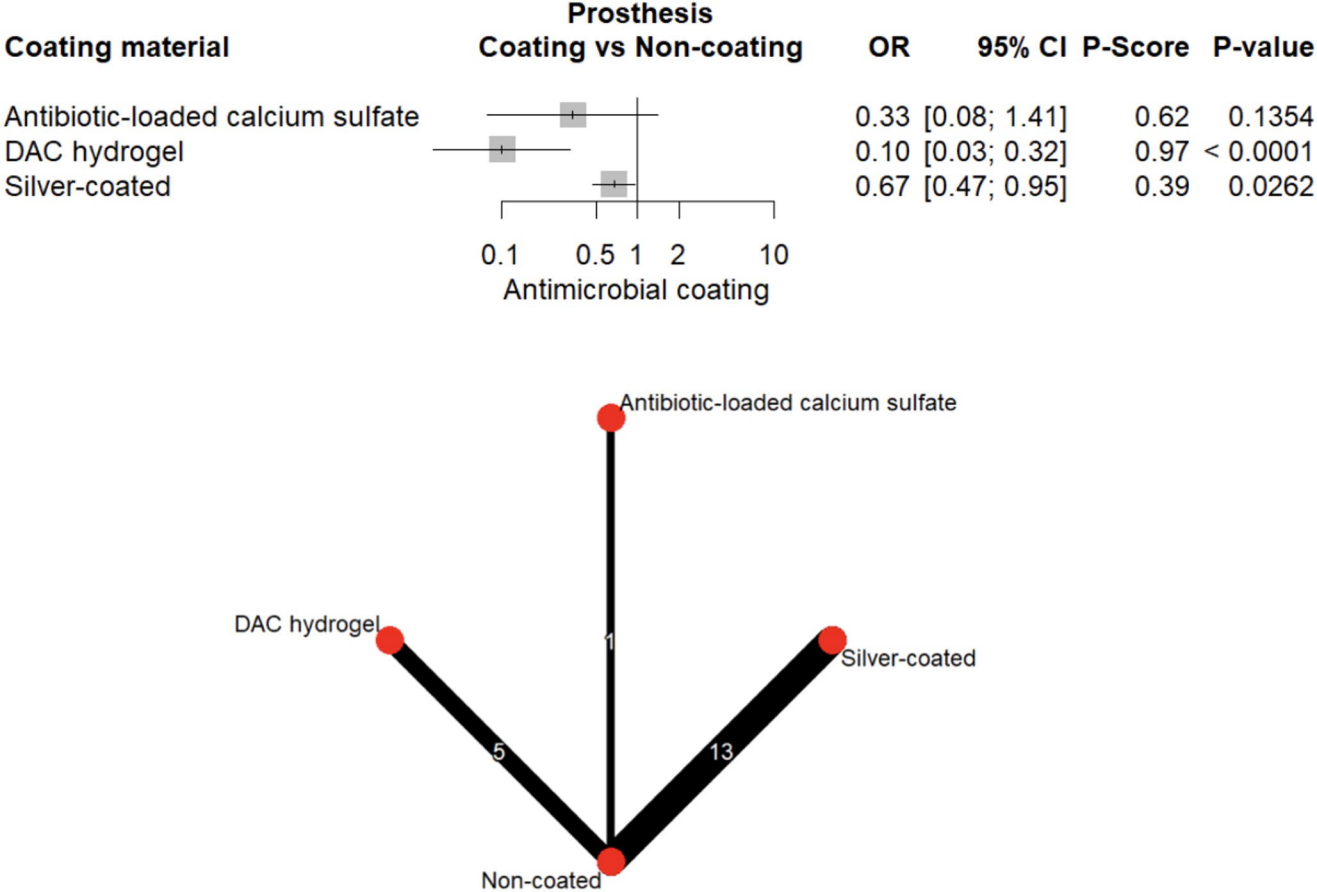
Forest plot and network graph illustrating the OR and 95% CIs for prosthetic joint infections, with ranked P-scores

**Fig. 10:**
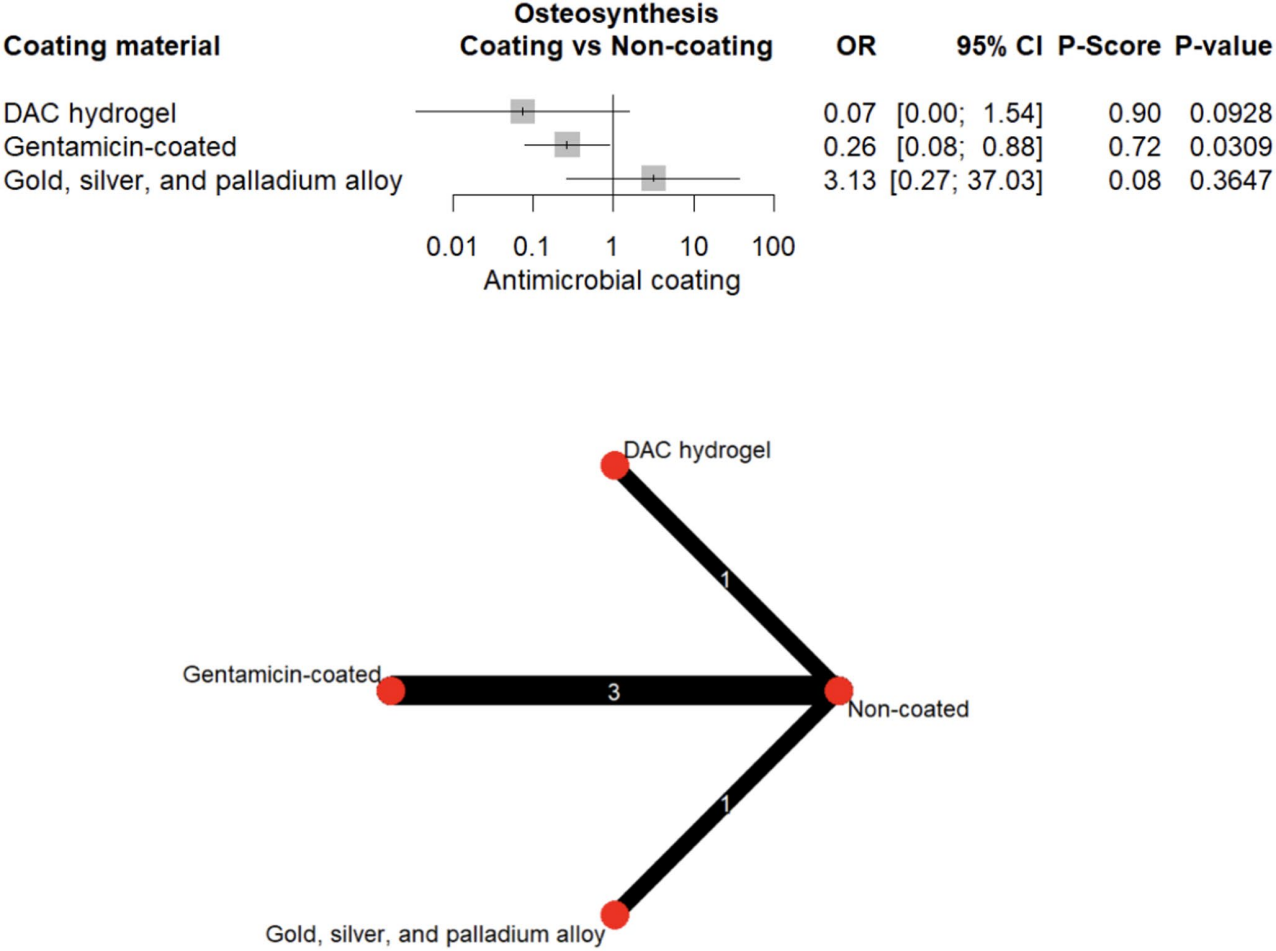
Forest plot and network graph illustrating the OR and 95% CIs for fracture-related infections, with ranked P-scores

When stratified by implant type, DAC-hydrogel (OR = 0.10, 95% CI: 0.03–0.32, *p* < 0.001) and silver (OR = 0.67, 95% CI: 0.47–0.95, *p* = 0.026) significantly reduced the odds of PJIs (Fig. 9). Gentamicin was similarly effective in reducing FRIs (OR = 0.26, 95% CI: 0.08–0.88, *p* = 0.031; Fig. 10). Evidence of small-study effects was minimal (Egger’s test, *p* = 0.030; Supplementary Fig. 3).

#### Infection outcomes

Among PJI patients, most required surgical intervention, primarily two-stage revision (15.6%) or debridement with implant retention (4.6%). Amputation was necessary in 10.1% of the cases, with a similar distribution between coated and uncoated implants. Infection resolution was reported in 6.64% of patients. Mortality was rare (1.4%) and showed no clear difference between groups.

#### Postoperative complications

**Fig. 11:**
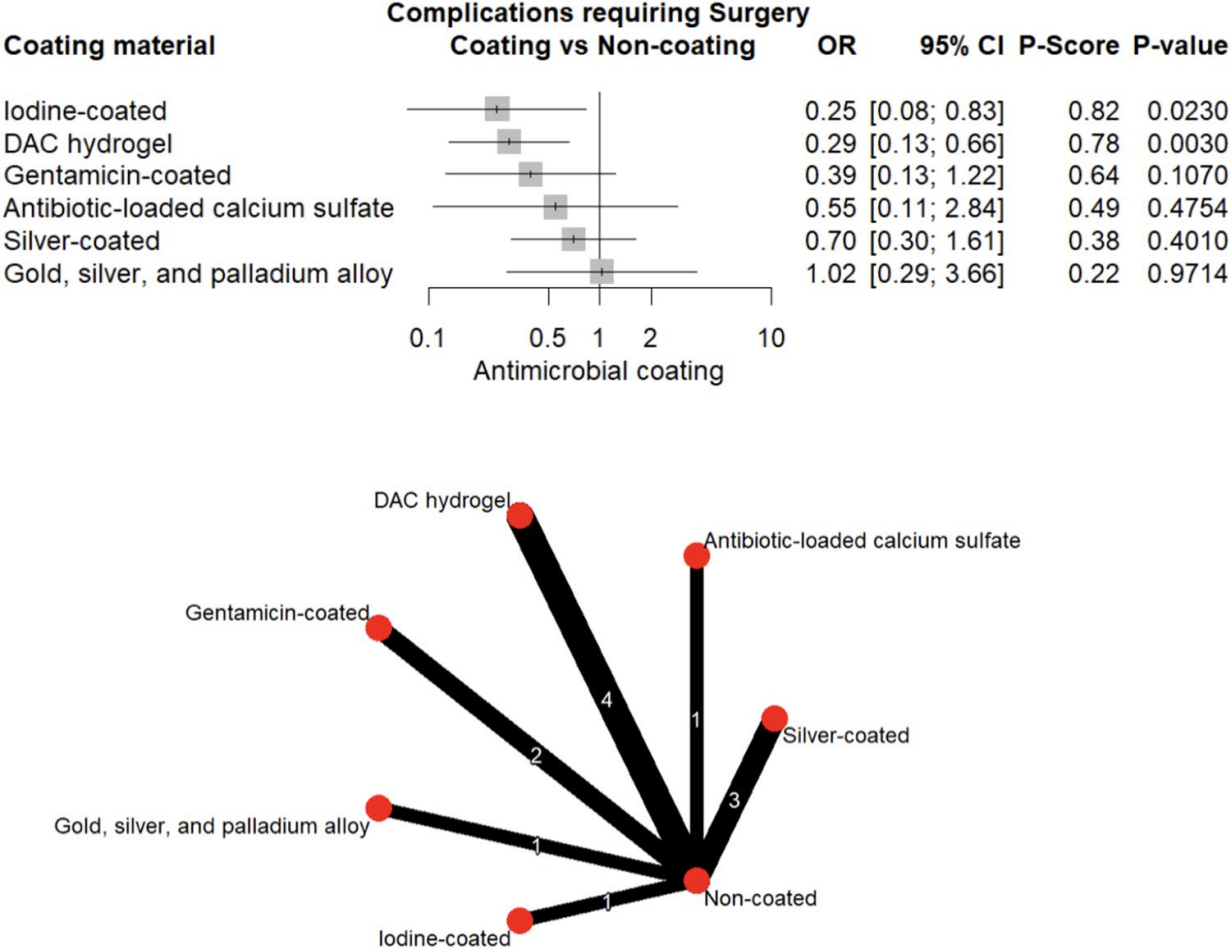
Forest plot and network graph illustrating the OR and 95% CIs for complications requiring surgery, with ranked P-scores

A total of 179 (9.7%) complications requiring surgical management were reported, occurring more frequently in the uncoated group (12.0%) than in the coated group (6.8%). The most common complications were delayed wound healing (4.5% vs. 9.5% for coated vs. uncoated) and delayed union (2.2% vs. 5.0%). Use of DAC-hydrogel (OR = 0.29, 95% CI: 0.13–0.66, *p* = 0.003) and iodine (OR = 0.25, 95% CI: 0.08–0.83, *p* = 0.023) was associated with significantly lower odds of postoperative complications (Fig. 11), with no evidence of publication bias (Egger’s test, *p* = 0.104; Supplementary Fig. 4).

#### Operative time

**Fig. 12:**
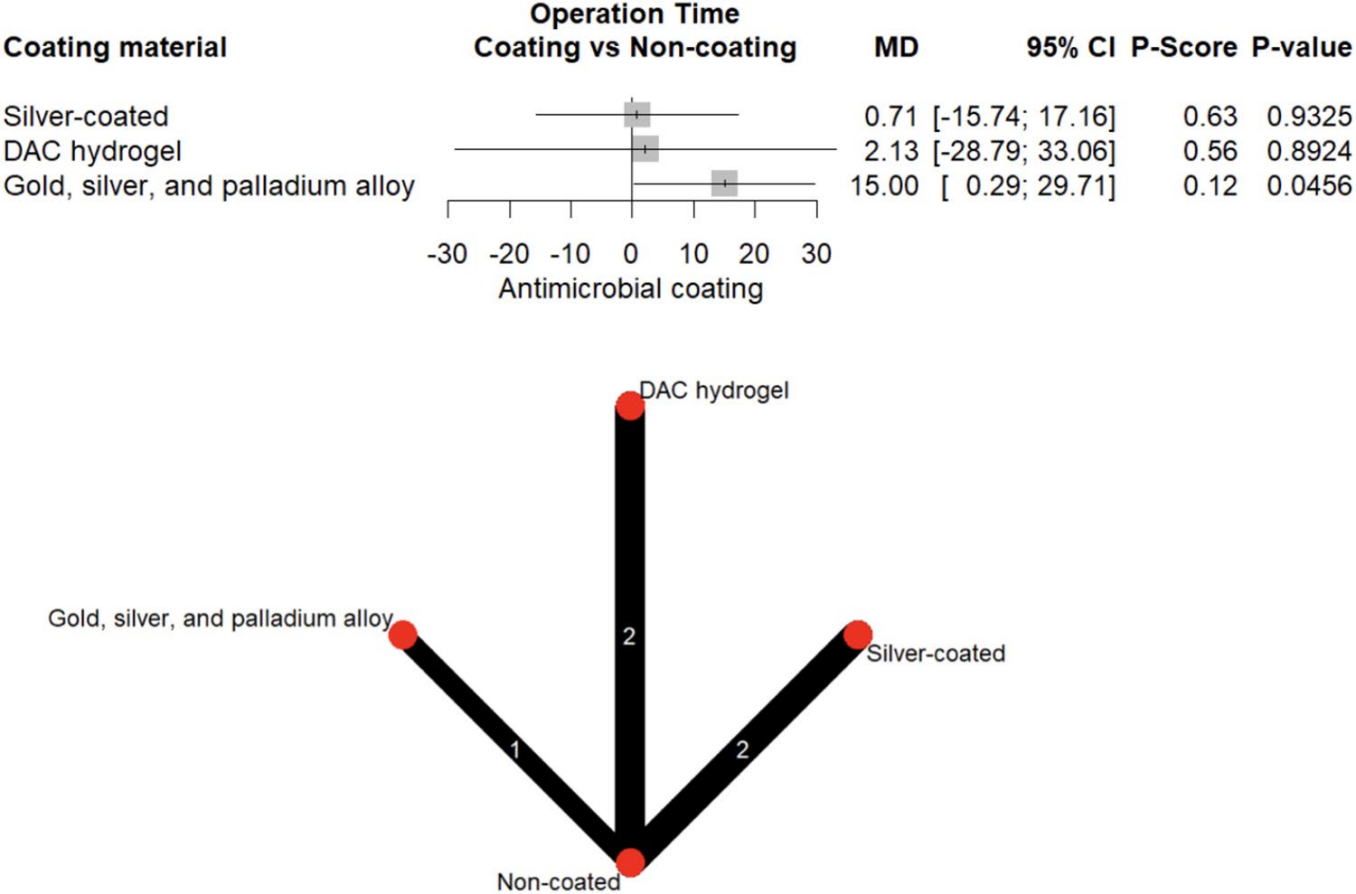
Forest plot and network graph illustrating the OR and 95% CIs for operative time, with ranked P-scores

The mean operative time was 85.0 ± 79.8 min for procedures performed with coated implants versus 76.0 ± 66.6 min for uncoated ones. Silver and DAC-hydrogel did not significantly prolong operative time (*p* > 0.05). In contrast, use of GSP alloy was associated with a modest but statistically significant increase in operative time (MD = 15.0 min, 95% CI: 0.30–29.71, *p* = 0.046; Fig. 12).

#### Perioperative antibiotic prophylaxis

**Fig. 13:**
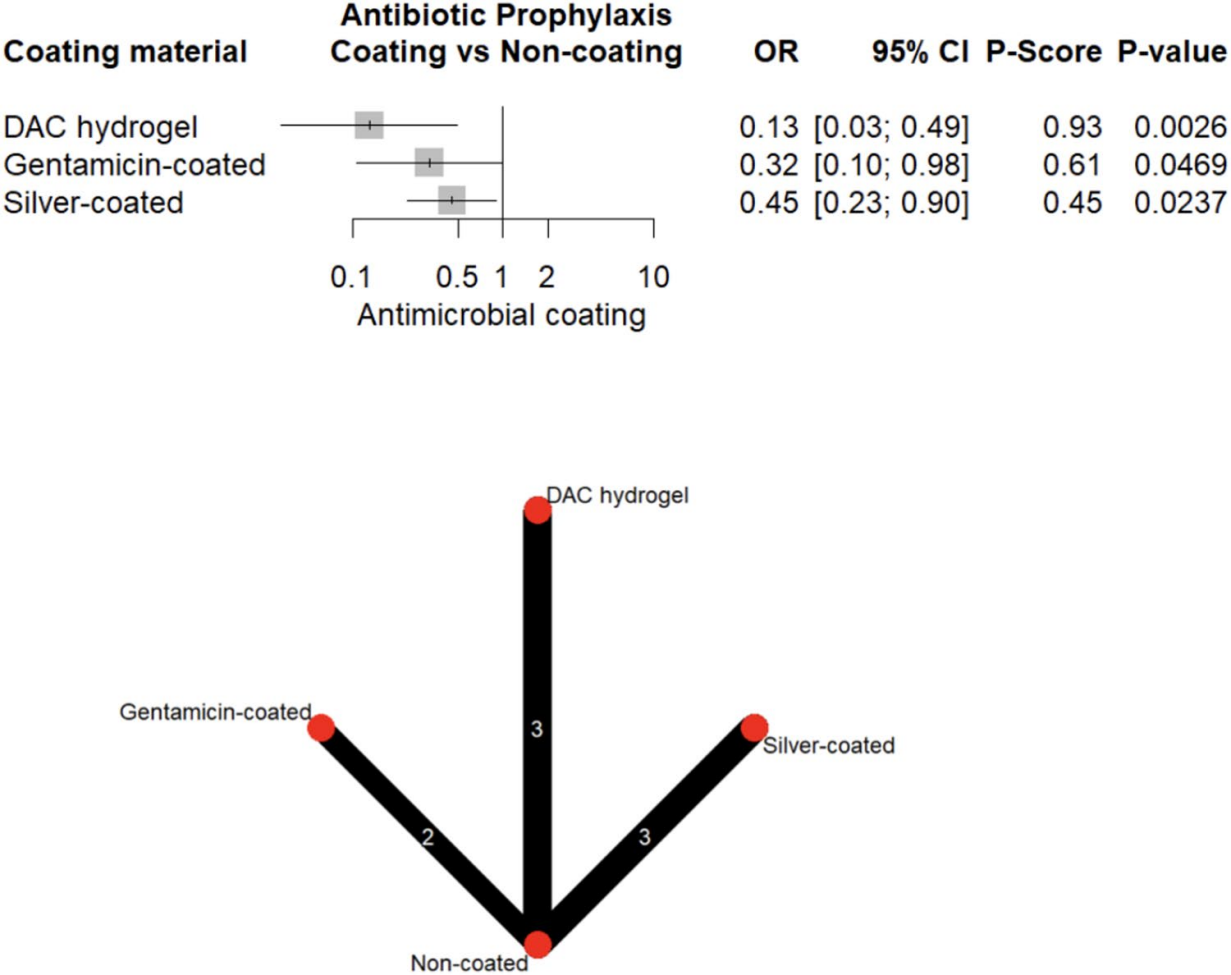
Forest plot and network graph illustrating the OR and 95% CIs for implant-associated infections occurring when perioperative antibiotic prophylaxis is administered, with ranked P-scores

**Fig. 14:**
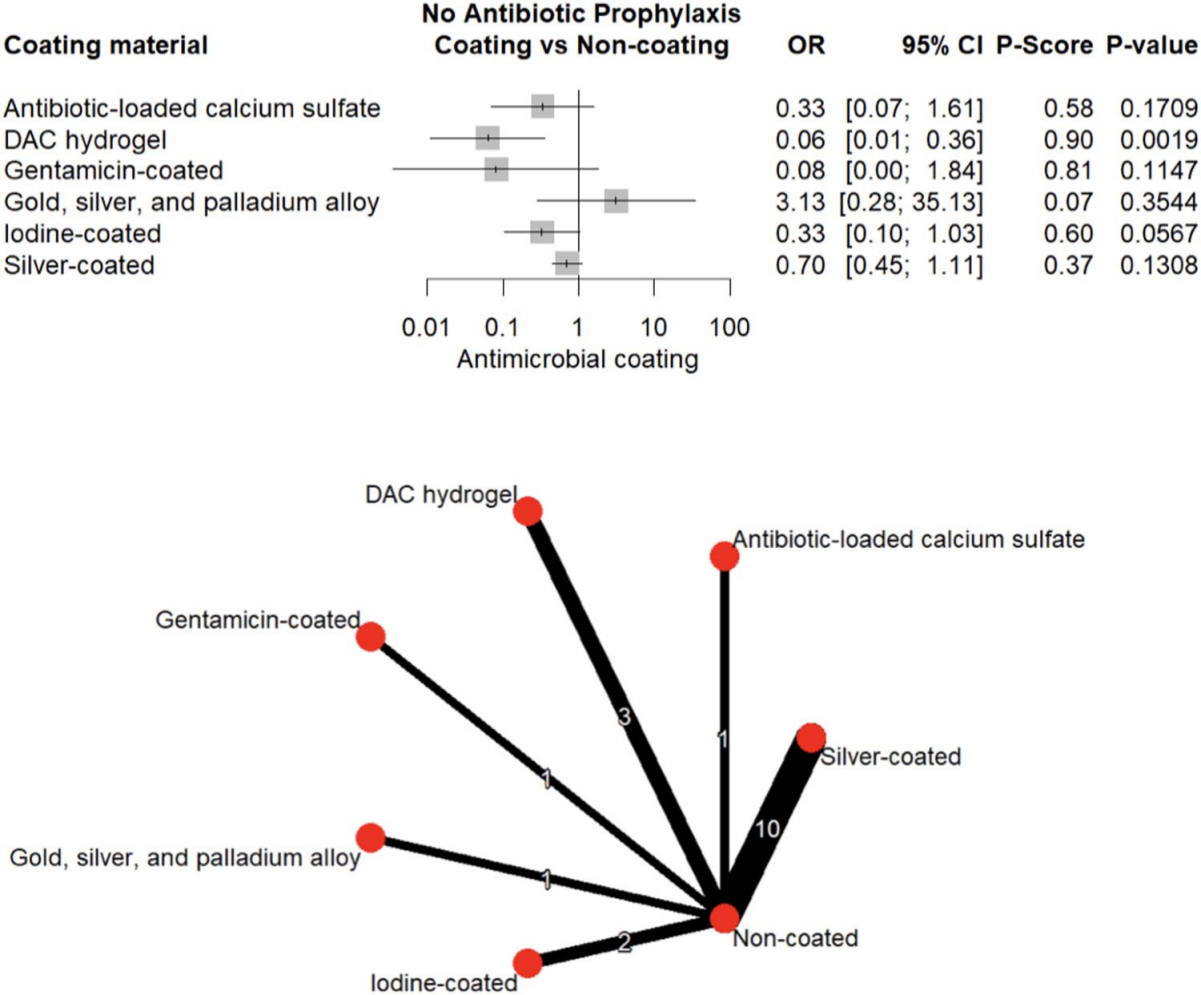
Forest plot and network graph illustrating the OR and 95% CIs for implant-associated infections occurring in absence of perioperative antibiotic prophylaxis, with ranked P-scores

1,103 patients (30.7%) received perioperative antibiotic prophylaxis; 560 (50.8%) in the coated and 543 (49.2%) in the uncoated group. Notably, in the absence of prophylaxis, gentamicin, iodine, and silver coatings did not significantly reduce infection risk (OR = 0.08–0.70, all *p* > 0.05) (Fig. 13). In contrast, DAC-hydrogel maintained a strong protective effect even when prophylactic antibiotics were not administered (OR = 0.06, 95% CI: 0.01–0.36, *p* = 0.001; Fig. 14).

#### Implant survival

Coated implants demonstrated slightly lower survival than uncoated implants over time. One-year survival was 91% for coated versus 95.1% for uncoated implants, while five-year survival was 84.5% versus 90.6%. At ten years, survival declined to 77.6% for coated implants and 70.0% for uncoated implants.

## Discussion

The most important finding of the present study is that while DAC-hydrogel, silver, iodine, and gentamicin were all associated with a significantly lower risk of infection than uncoated implants, their protective effect varied. LACs generally showed higher estimated effectiveness than other approaches, with DAC-hydrogel demonstrating the most favorable outcomes. These findings indicate that, within the limits of the current evidence base, LACs may represent the most consistently supported option for infection risk reduction.

The discrepancy in coating efficacy might be rooted in their distinct mechanisms of action. LACs, such as DAC-hydrogel and antibiotic-loaded calcium sulfate, are applied intraoperatively and deliver high local antibiotic concentrations at the implant-tissue interface, exceeding the minimal bactericidal concentration of common pathogens. This localized delivery prevents bacterial adhesion and early biofilm formation during the critical initial postoperative phase [60]. Antibiotic-loaded calcium sulfate may also contribute to bone regeneration by acting as an osteoconductive scaffold, and it serves as a temporary filler for dead space created during debridement, supporting both mechanical stability and infection control [61]. Conversely, ASMs provide sustained broad-spectrum antimicrobial effects through the controlled ion release and oxidative radical formation, which disrupt bacterial membranes and replication. While concerns regarding systemic silver toxicity persist, the doses reported in clinical studies are approximately 300 times lower than the established no-observed-adverse-effect level [28], and local or systemic argyrosis have not been reported [62]. This favorable safety profile likely reflects the therapeutic window for silver application, reported to be up to 10 µg/g [63]. It is important to recognize, however, that silver formulations differ markedly: elementary silver, commonly used in modular tumor endoprostheses, may involve silver loads of up to 3 g [62], whereas ionically active preparations such as Agluna^™^ release only around 6 mg, substantially reducing toxicity risk [28]. Finally, although bacterial resistance to silver has been documented, it remains uncommon and evolves more slowly than resistance to conventional antimicrobial agents [64].

Our findings are highly consistent with these pharmacological principles. The superior performance of LACs supports the hypothesis that achieving high-burst, supra-inhibitory local antibiotic concentrations might be the most effective strategy for eradicating bacteria during the decisive vulnerable window. Furthermore, while numerous in vitro studies have demonstrated silver’s strong protective activity [65–67], its clinical impact appears more variable, suggesting a lower in vivo estimated benefit. Similarly, the novel observation that ALCS does not reduce overall infection rates, but delays onset, aligns with its mechanism as a rapid-elution carrier, suggesting it provides effective, but transient, protection. While these trends support the preferential considerations of LACs, it is important to note that the evidence is largely observational and that interpretations should be seen as best-supported indications rather than definitive proof of clinical superiority.

A finding with potentially important clinical implications is the apparent efficacy of DAC-hydrogel in the absence of systemic antibiotic prophylaxis. While other coatings lost their protective effect when systemic antibiotics were withheld, the effect of DAC-hydrogel remained unaltered. This observation raises the hypothesis that LACs may, in selected settings, serve as an adjunct to, or possibly reduce reliance on, systemic administration. Despite the potential major implications for antibiotic stewardship, this interpretation should be regarded as hypothesis-generating and warrants further investigation.

Beyond infection prevention, coating technologies must be evaluated for their potential impact on osseointegration, mechanical stability, and long-term implant durability. Factors such as coating thickness, deposition method, and material properties can influence bone-implant interactions at load-bearing articular surfaces. These considerations are especially relevant in primary arthroplasty, where durable biological fixation is essential. In the present study, the observed lower long-term implant survival in the coated group likely reflects confounding by indication, as coated implants were preferentially used in higher-risk scenarios, such as revision procedures and tumor resections, which are inherently associated with higher mechanical failure. Consequently, these unadjusted observational data cannot be used to infer any causal relationship between antimicrobial coating and mechanical compromise.

From a health-economic standpoint, although antimicrobial coatings entail higher upfront costs, with an average direct cost ranging between €1,170 and €4,600, their use may generate downstream savings by reducing infection-related expenditures^68^. Trentinaglia et al. estimated that coatings achieving an 80% reduction in infection rates could result in hospital cost savings of approximately €200 per patient. In coatings offering lower levels of infection prevention, economic balance is maintained when use is limited to patients at high infection-risk [68]. In this context, the 90% reduction in infection odds observed with DAC-hydrogel suggests a favorable cost-effectiveness profile across a broad range of patient populations. These economic considerations remain preliminary, and formal cost-effectiveness analyses are needed to validate these projections. Notably, delayed adoption of coating technologies has been projected to incur an additional €440 million annually in infection-related healthcare costs, underscoring the potential economic relevance of timely adoption [69].

This study has several important limitations that must be acknowledged. First, the included data were largely unadjusted, introducing potential bias from confounding factors such as age, comorbidities, and antibiotic prophylaxis regimens. Without multivariate adjustment, the independent contribution of coating technologies cannot be conclusively verified, although subgroup network meta-analyses were conducted to mitigate this limitation. Second, silver coating was evaluated exclusively in prosthetic implants, limiting the generalizability of the findings to other implant types. While this is a known limitation of current silver-coating technologies [69], expanding their application to osteosynthesis devices could broaden their preventive potential. Finally, considerable heterogeneity was observed across the included studies with respect to design, follow-up duration, infection definition, and reporting standards. The lack of a standardized definition for IAIs remains a critical barrier to comparability. The analyzed studies variously applied MSIS [54], ICM [55], CDC [56], EBJIS [58], or unvalidated criteria, and previous evidence has demonstrated that infection detection rates vary substantially based on the used definition [70]. This heterogeneity likely contributes to variability in reported infection rates and limits the comparability of pooled estimates, emphasizing the need for adoption of a universal diagnostic framework, such as the 2021 EBJIS consensus criteria [58].

## Conclusion

There is enough statistical evidence to suggest that antimicrobial implant surface coatings reduce implant-associated infections and post-operative complications without increasing operative time. Local antibiotic carriers, including DAC-hydrogel and gentamicin, are associated with the largest reductions in infection risk, particularly in high-risk procedures. Surface-active coatings such as iodine and silver may confer additional, but more modest, benefit. Randomized trials are needed to further confirm the effectiveness, safety, optimal indications, and cost-utility of coating technologies.

## Supplementary Information

Below is the link to the electronic supplementary material.

Supplementary Material 1

## Data Availability

The datasets generated and analyzed during the current study are available from the corresponding author on reasonable request.

## Abbreviations

IAIs: Implant-associated infections
PSM: Passive surface modification
ASM: Active surface modification
LAC: Local antibiotic carrier
DAC: Defensive antibacterial coating
ALCS: Antibiotic-loaded calcium sulfate
PJI: Prosthetic joint infection
FRI: Fracture-related infection
EPS: Extracellular polymeric substance
PMMA: Polymethylmethacrylate
PRISMA: Preferred reporting items for systematic reviews and meta-analysis
ROB 2: Revised cochrane risk-of-bias tool
ROBINS-I: Risk of bias in non-randomized studies – of intervention
MSIS: Musculoskeletal infection society
ICM: International consensus meeting
CDC: Centers for disease control and prevention
EBJIS: European bone and joint infection society
PDDLA: Poly D, L-lactide
GSP: Gold, silver, palladium
