# Supplementary material for: Comparative effectiveness of antimicrobial implant surface coatings in preventing orthopaedic implant-associated infections: a network meta-analysis
Source: Arch Orthop Trauma Surg. 2026 Feb 10;146(1):60. doi: 10.1007/s00402-026-06225-3 (PMC12891049; doi:10.1007/s00402-026-06225-3)
Supplement: Supplementary file 1 — Supplementary Material 1 [file 402_2026_6225_MOESM1_ESM.docx]

**
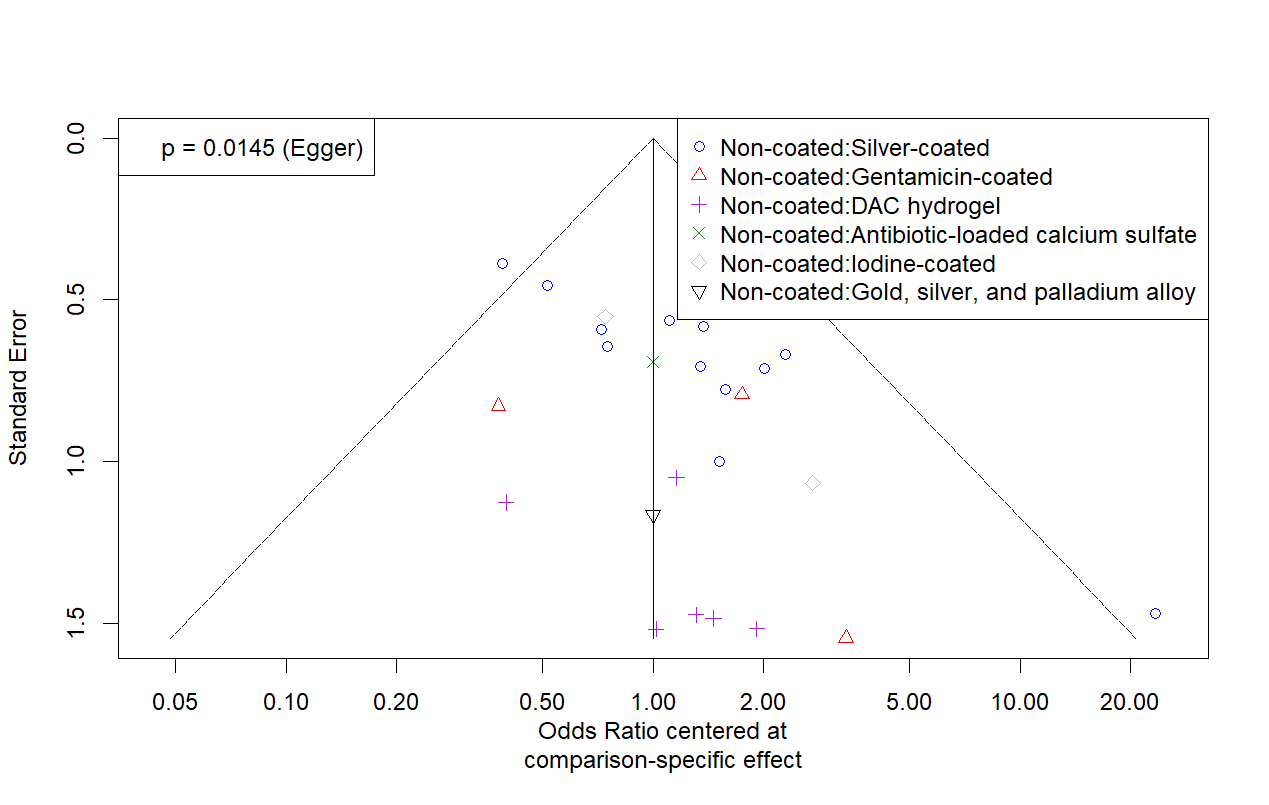
**Supplementary Figure 1. Funnel plot with Egger’s regression test for implant-associated infections.

**
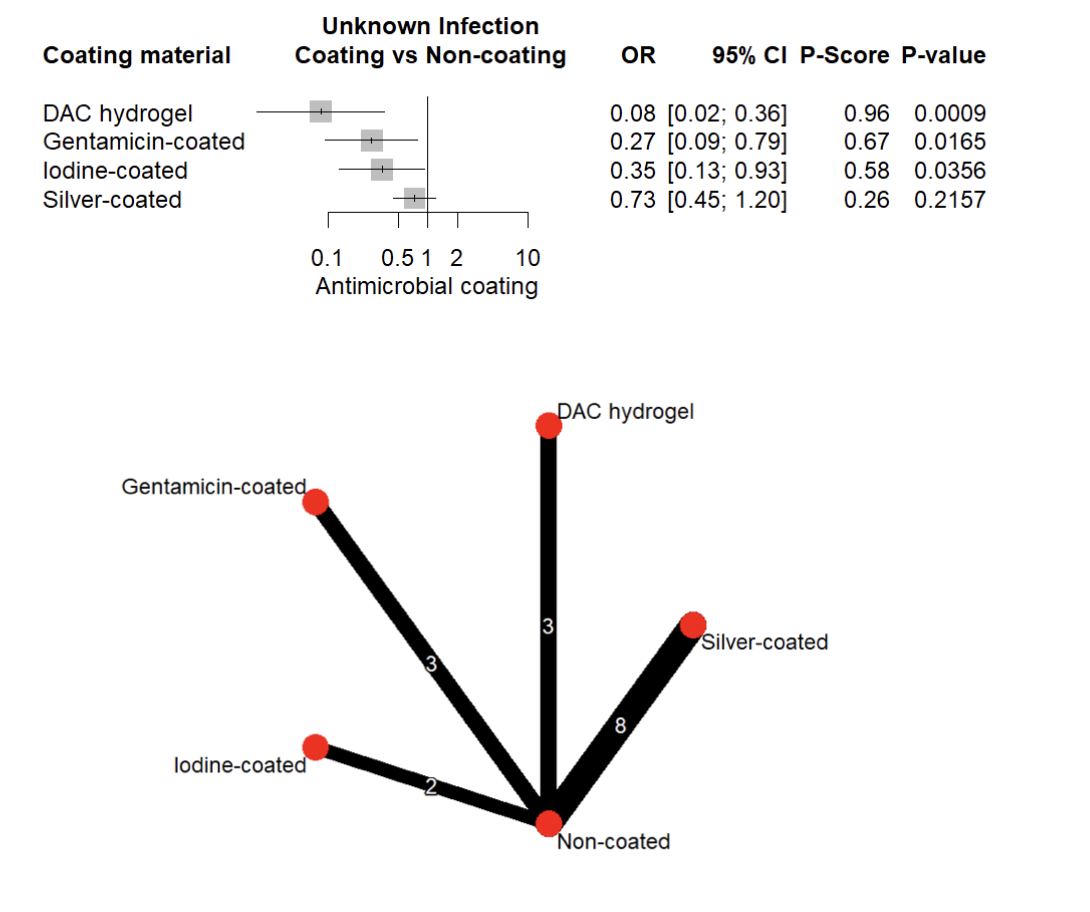
**

Supplementary Figure 2. Forest plot and network graph illustrating the OR and 95% CIs for infections of unknown origin, with ranked P-scores.


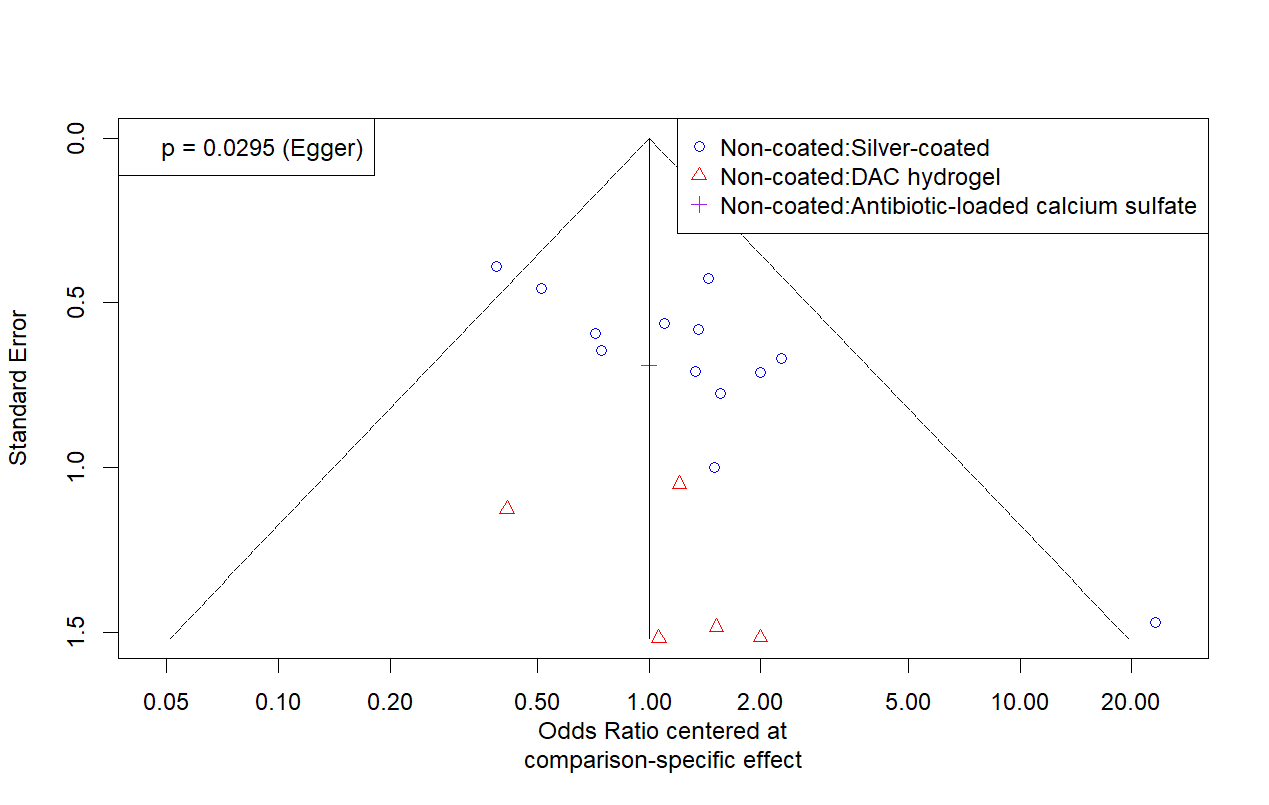
Supplementary Figure 3. Funnel plot with Egger’s regression test for prosthesis joint infections.

**
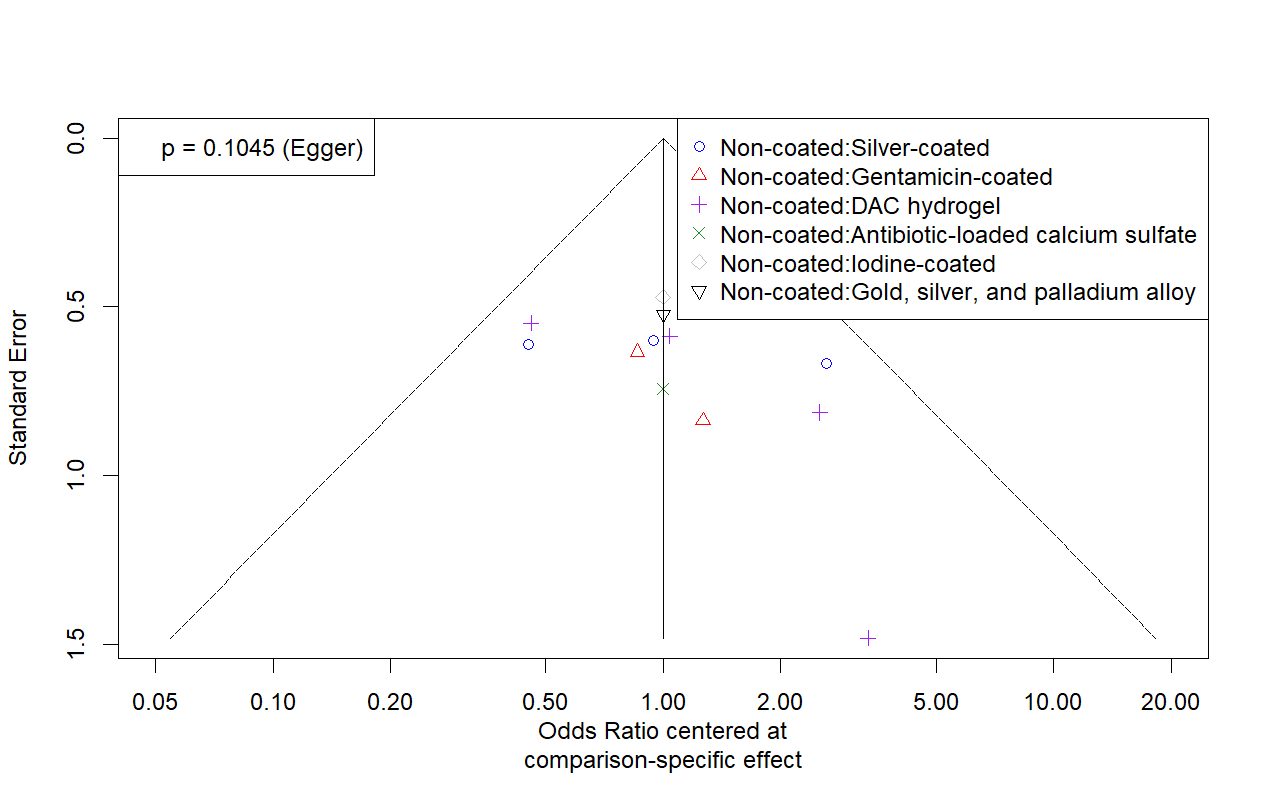
**Supplementary Figure 4. Funnel plot with Egger’s regression test for complications requiring surgery.

Supplementary Table 1. Summary of the search strategy used for literature search in the four databases.

| **Search terms** |
| --- |
| **Ovid Medline**   1. exp Coated Materials, Biocompatible/ 2. ((silver or iodine or hydroxyapatite or hydrogel or DAC or nanomaterial or nanoparticle or antibiotic or gentamicin or clindamycin or vancomycin or tobramycin or rifampicin or cefazolin or daptomycin or linezolid or antibacterial or antimicrobial) and (coat* or cover* or layer* or plat*)).mp. 3. 1 or 2 4. “prostheses and implants”/ or exp bone-anchored prosthesis/ or exp bone-implant interface/ or exp joint prosthesis/ 5. (implant or nail or plate or screw or wire or fixator or Ilizarov or osteosynthesis or graft or prosthesis or megaprosthes* or endoprosthes* or “joint replacement” or “knee implant” or “hip implant” or arthroplasty).mp. 6. 4 or 5 7. exp Prosthesis-Related Infections/ 8. (“implant-related infection” or “implant infection” or “surgical site infection” or “biofilm infection” or “deep surgical infection” or “periprosthetic joint infection” or “periprosthetic infection” or “device-associated infection”).mp 9. 7 or 8 10. 3 and 6 and 9 |
| **Embase**   1. exp material coating/ 2. ((silver or iodine or hydroxyapatite or hydrogel or DAC or nanomaterial or nanoparticle or antibiotic or gentamicin or clindamycin or vancomycin or tobramycin or rifampicin or cefazolin or daptomycin or linezolid or antibacterial or antimicrobial) and (coat* or cover* or layer* or plat*)).mp. 3. 1 or 2 4. exp bone implant/ 5. (implant or nail or plate or screw or wire or fixator or Ilizarov or osteosynthesis or graft or prosthesis or megaprosthes* or endoprosthes* or "joint replacement" or "knee implant" or "hip implant" or arthroplasty or pin).mp. 6. 4 or 5 7. exp prosthesis infection/ or exp periprosthetic joint infection/ 8. ("implant-related infection" or "implant infection" or "surgical site infection" or "biofilm infection" or "deep surgical infection" or "periprosthetic joint infection" or "periprosthetic infection" or "device-associated infection").mp. 9. 7 or 8 10. 3 and 6 and 9 |
| **Scopus**  TITLE-ABS-KEY((silver OR iodine OR hydroxyapatite OR hydrogel OR dac OR nanomaterial OR nanoparticle OR antibiotic OR gentamicin OR clindamycin OR vancomycin OR tobramycin OR rifampicin OR cefazolin OR daptomycin OR linezolid OR antibacterial OR antimicrobial) AND (coat* OR cover* OR layer* OR plat*) AND (implant OR nail OR plate OR screw OR wire OR fixator OR ilizarov OR osteosynthesis OR graft OR prosthesis OR megaprosthes* OR endoprosthes* OR "joint replacement" OR "knee implant" OR "hip implant" OR arthroplasty OR pin) AND ("implant-related infection" OR "implant infection" OR "surgical site infection" OR "biofilm infection" OR "deep surgical infection" OR "periprosthetic joint infection" OR "periprosthetic infection" OR "device-associated infection")) |
| **Web of Science**  ((TS=(silver OR iodine OR hydroxyapatite OR hydrogel OR dac OR nanomaterial OR nanoparticle OR antibiotic OR gentamicin OR clindamycin OR vancomycin OR tobramycin OR rifampicin OR cefazolin OR daptomycin OR linezolid OR antibacterial OR antimicrobial ) AND ( coat* OR cover* OR layer* OR plat* ) AND ( implant OR nail OR plate OR screw OR wire OR fixator OR ilizarov OR osteosynthesis OR graft OR prosthesis OR megaprosthes* OR endoprosthes* OR "joint replacement" OR "knee implant" OR "hip implant" OR arthroplasty OR pin ) AND ( "implant-related infection" OR "implant infection" OR "surgical site infection" OR "biofilm infection" OR "deep surgical infection" OR "periprosthetic joint infection" OR "periprosthetic infection" OR "device-associated infection"))) |

Supplementary Table 2. Number, site, and microbiological profile of implant-associated infections reported by the included studies.

| **Author, Year** | **IAIs** | | | | | | | | | |
| --- | --- | --- | --- | --- | --- | --- | --- | --- | --- | --- |
|  | **Total** | **Coated** | **Uncoated** | **Early** | **Late** | **Isolated micro-organisms** | **Gram +ve** | **Gram -ve** | **Mixed culture** | **Culture negative** |
| **Silver** | | | | | | | | | | |
| Hardes et al., 2010^1^ | 16 | 3 (5.8%) | 13 (17.5%) | NR | NR | *S. pyogenes, S. hemolyticus, S. epidermidis, Enterococcus faecalis, S. hominis* | 10 (62.5%) | 0 (0%) | 0 (0%) | 4 (25%) |
| Wafa et al., 2015^2^ | 29 | 10 (11.7%) | 19 (22.3%) | NR | NR | *S. aureus, S. viridans, E. faecalis, beta-hemolytic streptococci, P. aeruginosa, Enterobacter cloacae, E. coli, Proteus mirabilis, Brevundimonas vesicularis, Raoultella planticola* | 24 (82.7%)^*^ | 10 (34.5%)^*^ | 0 (0%) | 3 (10.3%) |
| Donati et al., 2016^3^ | 8 | 3 (8%) | 5 (16.6%) | 4 (50%) | 4 (50%) | NR | NR | NR | NR | NR |
| Piccioli et al., 2016^4^ | 5 | 2 (11.7%) | 3 (23%) | 2 (40%) | 3 (60%) | NR | NR | NR | NR | NR |
| Hardes et al., 2017^5^ | 15 | 7 (12.5%) | 8 (19%) | NR | NR | *S. aureus, P. aeruginosa,* MRSA*, S. pyogenes, S. hemolyticus, S. lugdunensis, S. caprae, S. agalactiae, S. epidermidis* | 11 (73.3%) | 1 (6.6%) | 0 (0%) | 0 (0%) |
| Zajonz et al., 2017^6^ | 16 | 8 (40%) | 8 (57.1%) | NR | NR | NR | NR | NR | NR | NR |
| Medellin et al., 2019^7^ | 15 | 4 (17.4%) | 11 (19%) | NR | NR | NR | NR | NR | NR | NR |
| Parry et al., 2019^8^ | 34 | 11 (12.3%) | 23 (7.5%) | NR | NR | CNS, *S. aureus, E. cloacae, E. coli*, MRSA, MSSA, *S. viridans*, flucloxacillin-resistant CNS, multidrug-resistant CNS, *Corynebacterium spp*., vancomycin-resistant enterococcus, *P. aeruginosa* | 27 (79.4%) | 1 (3%) | 1 (3%) | 5 (14.7%) |
| Sambri et al., 2019^9^ | 16 | 0 (0%) | 16 (45.7%) | NR | NR | NR | NR | NR | NR | NR |
| Streitbuerger et al., 2019^10^ | 14 | 7 (9.4%) | 7 (20%) | NR | NR | *S. aureus, E. faecalis, S. epidermidis, S. angionosus, S. constellatus, S. hominis* | 10 (71.4%) | 0 (0%) | NR | NR |
| Sambri et al., 2020^11^ | 13 | 3 (10.3%) | 10 (25.6%) | NR | NR | NR | NR | NR | NR | NR |
| Pala et al., 2022^12^ | 13 | 8 (6.8%) | 5 (7.2%) | NR | NR | NR | NR | NR | NR | NR |
| Sacchetti et al., 2022^13^ | 29 | 9 (23.7%) | 20 (19.2%) | NR | NR | NR | NR | NR | NR | NR |
| **DAC-hydrogel** | | | | | | | | | | |
| Romano et al., 2016^14^ | 12 | 1 (0.5%) | 11 (6%) | NR | NR | NR | NR | NR | NR | NR |
| Malizos et al., 2017^15^ | 6 | 0 (0%) | 6 (4.7%) |  |  | NR | NR | NR | NR | NR |
| Zagra et al., 2019^16^ | 4 | 0 (0%) | 4 (14.8%) | NR | NR | MRSA, *S. epidermidis, S. capitis* | NR | NR | NR | NR |
| De Meo et al., 2020^17^ | 6 | 0 (0%) | 6 (35.3%) | NR | NR | MRSA, MSSA, *E. faecalis, Klebsiella pneumoniae, E. coli* | 5 (83.3%) | 0 (0%) | 0 (0%) | 1 (16.6%) |
| Zoccali et al., 202^18^ | 6 | 0 (0%) | 6 (14%) | NR | NR | NR | NR | NR | NR | 1 (16.6%) |
| Ding et al., 2025^19^ | 5 | 1 | 4 | NR | NR | *S. aureus,* MRSA*, S. capitis* | 5 | 0 | 0 | 0 |
| **Gentamicin** | | | | | | | | | | |
| Pinto et al., 2019^20^ | 4 | 0 (0%) | 4 (28.6%) | NR | NR | NR | NR | NR | NR | NR |
| Greco et al., 2021^21^ | 7 | 3 (13%) | 4 (17.4%) | NR | NR | NR | NR | NR | NR | NR |
| Rai et al., 2025^22^ | 13 | 2 (3.2%) | 11 (17.7%) | 13 (100%) | 0 (0%) | NR | NR | NR | NR | NR |
| **Iodine** | | | | | | | | | | |
| Shirai et al., 2016^23^ | 12 | 1 (2.6%) | 11 (17.7%) | NR | NR | NR | NR | NR | NR | NR |
| Miwa et al., 2019^24^ | 33 | 4 (6%) | 29 (12.3%) | NR | NR | NR | NR | NR | NR | NR |
| **Gold, silver, palladium alloy** | | | | | | | | | | |
| Wiechert et al., 2025^25^ | 4 | 3 (3.2%) | 1 (1%) | NR | NR | *E. cloacae, E. faecalis, E. kobei, cutibacterium acnes, bacteroides fragilis, E. coli, S. epidermidis, S. aureus* | 3 (75%) | 0 (0%) | 1 (25%) | 0 (0%) |
| **Antibiotic-loaded calcium sulphate** | | | | | | | | | | |
| McPherson et al., 2024^26^ | 11 | 3 (2.7%) | 8 (7.7%) | NR | NR | MRSA, *S. epidermidis, E. faecalis, S. hominis, vancomycin-resistant Enterococcus spp., Ochrobactrum anthropi, S. agalactiae, Klebsiella pneumoniae* | 8 (72.7%) | 0 (0%) | 2 (18.2%) | 1 (9.1%) |

*Polymicrobial infections were detected. CNS: coagulase-negative Staphylococcus; MRSA: methicillin-resistant Staphylococcus aureus; MSSA: methicillin-sensitive Staphylococcus aureus; NR: Not reported.

**References**

1. Hardes J, von Eiff C, Streitbuerger A, Balke M, Budny T, Henrichs MP, et al. Reduction of periprosthetic infection with silver-coated megaprostheses in patients with bone sarcoma. J Surg Oncol. 2010 Apr 1;101(5):389-95.

2. Wafa H, Grimer RJ, Reddy K, Jeys L, Abudu A, Carter SR, et al. Retrospective evaluation of the incidence of early periprosthetic infection with silver-treated endoprostheses in high-risk patients: case-control study. Bone Joint J. 2015 Feb;97-b(2):252-7.

3. Donati F, Di Giacomo G, D'Adamio S, Ziranu A, Careri S, Rosa M, et al. Silver-Coated Hip Megaprosthesis in Oncological Limb Savage Surgery. Biomed Res Int. 2016;2016:9079041. Epub 20160823.

4. Piccioli A, Donati F, Giacomo GD, Ziranu A, Careri S, Spinelli MS, et al. Infective complications in tumour endoprostheses implanted after pathological fracture of the limbs. Injury. 2016 Oct;47 Suppl 4:S22-s8. Epub 20160825.

5. Hardes J, Henrichs MP, Hauschild G, Nottrott M, Guder W, Streitbuerger A. Silver-Coated Megaprosthesis of the Proximal Tibia in Patients With Sarcoma. J Arthroplasty. 2017 Jul;32(7):2208-13. Epub 20170301.

6. Zajonz D, Birke U, Ghanem M, Prietzel T, Josten C, Roth A, et al. Silver-coated modular Megaendoprostheses in salvage revision arthroplasty after periimplant infection with extensive bone loss - a pilot study of 34 patients. BMC Musculoskelet Disord. 2017 Sep 2;18(1):383. Epub 20170902.

7. Medellin MR, Fujiwara T, Clark R, Stevenson JD, Parry M, Jeys L. Mechanisms of failure and survival of total femoral endoprosthetic replacements. Bone Joint J. 2019 May;101-b(5):522-8.

8. Parry MC, Laitinen MK, Albergo JI, Gaston CL, Stevenson JD, Grimer RJ, et al. Silver-coated (Agluna®) tumour prostheses can be a protective factor against infection in high risk failure patients. Eur J Surg Oncol. 2019 Apr;45(4):704-10. Epub 20181214.

9. Sambri A, Bianchi G, Parry M, Frenos F, Campanacci D, Donati D, et al. Is Arthrodesis a Reliable Salvage Option following Two-Stage Revision for Suspected Infection in Proximal Tibial Replacements? A Multi-Institutional Study. J Knee Surg. 2019 Sep;32(9):911-8. Epub 20180918.

10. Streitbuerger A, Henrichs MP, Hauschild G, Nottrott M, Guder W, Hardes J. Silver-coated megaprostheses in the proximal femur in patients with sarcoma. Eur J Orthop Surg Traumatol. 2019 Jan;29(1):79-85. Epub 20180620.

11. Sambri A, Zucchini R, Giannini C, Zamparini E, Viale P, Donati DM, et al. Silver-coated (PorAg(®)) endoprosthesis can be protective against reinfection in the treatment of tumor prostheses infection. Eur J Orthop Surg Traumatol. 2020 Dec;30(8):1345-53. Epub 20200524.

12. Pala E, Trovarelli G, Ippolito V, Berizzi A, Ruggieri P. A long-term experience with Mutars tumor megaprostheses: analysis of 187 cases. Eur J Trauma Emerg Surg. 2022 Jun;48(3):2483-91. Epub 20211102.

13. Sacchetti F, Kilian R, Muratori F, Cherix S, Foschi L, Morganti R, et al. The Performances of Conventional Titanium and Silver-Coated Megaprostheses in Non-oncological and Post-oncological Patients: An Analysis of Infection Failures in 142 Patients. Arch Bone Jt Surg. 2022 May;10(5):439-46.

14. Romanò CL, Malizos K, Capuano N, Mezzoprete R, D'Arienzo M, Van Der Straeten C, et al. Does an Antibiotic-Loaded Hydrogel Coating Reduce Early Post-Surgical Infection After Joint Arthroplasty? J Bone Jt Infect. 2016;1:34-41. Epub 20160719.

15. Malizos K, Blauth M, Danita A, Capuano N, Mezzoprete R, Logoluso N, et al. Fast-resorbable antibiotic-loaded hydrogel coating to reduce post-surgical infection after internal osteosynthesis: a multicenter randomized controlled trial. J Orthop Traumatol. 2017 Jun;18(2):159-69. Epub 20170202.

16. Zagra L, Gallazzi E, Romanò D, Scarponi S, Romanò C. Two-stage cementless hip revision for peri-prosthetic infection with an antibacterial hydrogel coating: results of a comparative series. Int Orthop. 2019 Jan;43(1):111-5. Epub 20181030.

17. De Meo D, Calogero V, Are L, Cavallo AU, Persiani P, Villani C. Antibiotic-Loaded Hydrogel Coating to Reduce Early Postsurgical Infections in Aseptic Hip Revision Surgery: A Retrospective, Matched Case-Control Study. Microorganisms. 2020 Apr 15;8(4). Epub 20200415.

18. Zoccali C, Scoccianti G, Biagini R, Daolio PA, Giardina FL, Campanacci DA. Antibacterial hydrogel coating in joint mega-prosthesis: results of a comparative series. Eur J Orthop Surg Traumatol. 2021 Dec;31(8):1647-55. Epub 20210205.

19. Ding BTK, D'Apolito R, Sciamanna L, Zagra L. Antibacterial hydrogel coating is associated with lower complication risks after complex high-risk primary and cementless hip revision arthroplasty : a retrospective matched cohort study. Bone Jt Open. 2025 Jun 1;6(6 Supple B):15-23. Epub 20250601.

20. Pinto D, Manjunatha K, Savur AD, Ahmed NR, Mallya S, Ramya V. Comparative study of the efficacy of gentamicin-coated intramedullary interlocking nail versus regular intramedullary interlocking nail in Gustilo type I and II open tibia fractures. Chin J Traumatol. 2019 Oct;22(5):270-3. Epub 20190621.

21. Greco T, Cianni L, Polichetti C, Inverso M, Maccauro G, Perisano C. Uncoated vs. Antibiotic-Coated Tibia Nail in Open Diaphyseal Tibial Fracture (42 according to AO Classification): A Single Center Experience. Biomed Res Int. 2021;2021:7421582. Epub 20211014.

22. Rai SK, Gupta TP, Kashid M, Sirohi B, Kale A, Sharma R, et al. Does a gentamicin-coated intramedullary nail prevent postoperative infection in Gustilo type I and II tibial open fractures? A comparative study and retrospective analysis. Eur J Trauma Emerg Surg. 2025 Jan 27;51(1):86. Epub 20250127.

23. Shirai T, Tsuchiya H, Terauchi R, Tsuchida S, Mizoshiri N, Igarashi K, et al. The outcomes of reconstruction using frozen autograft combined with iodine-coated implants for malignant bone tumors: compared with non-coated implants. Jpn J Clin Oncol. 2016 Aug;46(8):735-40. Epub 20160520.

24. Miwa S, Shirai T, Yamamoto N, Hayashi K, Takeuchi A, Tada K, et al. Risk factors for surgical site infection after malignant bone tumor resection and reconstruction. BMC Cancer. 2019 Jan 8;19(1):33. Epub 20190108.

25. Wiechert J, Osterhoff G, Kleber C, Höch A, Notov D. Safety and complications of antimicrobial coated compared to conventional intramedullary femoral nails in proximal femoral fractures. Eur J Trauma Emerg Surg. 2025 Mar 12;51(1):132. Epub 20250312.

26. McPherson EJ, Crawford BM, Kenny SG, Dipane MV, Salarkia S, Stavrakis AI, et al. Point-of-Care Coating of Revision Femoral Stems With Antibiotic-Loaded Calcium Sulfate: Reduction in Infection After 2nd Stage Reimplantation but Not With Aseptic Revisions. Arthroplast Today. 2024 Feb;25:101302. Epub 20240122.
